# Potential of Fiber and Probiotics to Fight Against the Effects of PhIP + DSS-Induced Carcinogenic Process of the Large Intestine

**DOI:** 10.1021/acs.jafc.4c07366

**Published:** 2024-10-29

**Authors:** Aida Zapico, Nuria Salazar, Silvia Arboleya, Carmen González del Rey, Elena Diaz, Ana Alonso, Miguel Gueimonde, Clara G. de los Reyes-Gavilán, Celestino Gonzalez, Sonia González

**Affiliations:** † Department of Functional Biology, University of Oviedo, Oviedo 33006, Spain; ‡ Department of Microbiology and Biochemistry of Dairy Products, Instituto de Productos Lácteos de Asturias (IPLA-CSIC), Villaviciosa 33300, Spain; § Anatomical Pathology Service, Central University Hospital of Asturias (HUCA), Oviedo 33011, Spain; ∥ Diet, Microbiota and Health Group, Instituto de Investigación Sanitaria del Principado de Asturias (ISPA), Oviedo 33011, Spain

**Keywords:** fiber, PhIP, probiotic, microbiota, colorectal cancer, treatment strategies, heterocyclic amines, xenobiotics

## Abstract

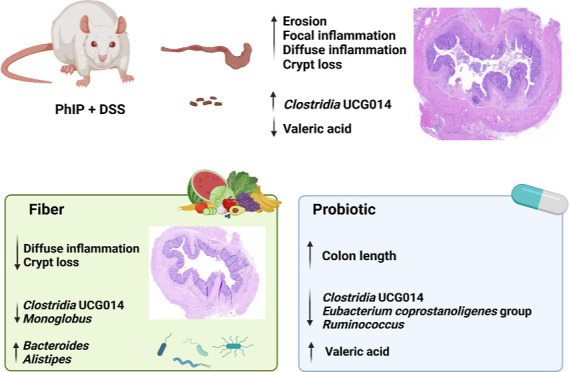

We determined the in vivo counteracting effect of fiber and probiotic supplementation on colonic mucosal damage and alterations in gut microbiota caused by 2-amino-1-methyl-6-phenylimidazo [4,5-*b*] pyridine (PhIP) and sodium dextran sulfate (DSS). Male Fischer-344 rats were randomly divided into 4 groups: control (standard diet), PhIP + DSS group (standard diet + PhIP + DSS), fiber (fiber diet + PhIP + DSS), and probiotic (probiotic diet + PhIP + DSS). The intake of PhIP + DSS for 3 weeks induced colonic mucosal erosion, crypt loss, and inflammation, and the distal colon was more severely damaged. Fiber alleviated colonic mucosal damage by reducing crypt loss and inflammation, while the probiotic increased colon length. The intake of PhIP + DSS increased the fecal relative abundance of *Clostridia* UCG014 along the intervention, in contrast to the lower abundances of these taxa found after PhIP + DSS administration in the rats supplemented with probiotics or fiber. Fiber supplementation mitigated the histological damage caused by PhIP + DSS shifting the gut microbiota toward a reduction of pro-inflammatory taxa.

## Introduction

Epidemiologic studies have shown that diet is among the most important environmental factors contributing to the development of cancer in humans. Dietary exposure to carcinogens depends on the type of food, its preparation, and nutrient composition.^1^ In particular, heterocyclic aromatic amines (HCAs) are formed mainly by the pyrolysis of aromatic amino acids and creatine from protein-containing foods during cooking at high temperatures and have shown a high mutagenic potential.^2^ 2-Amino-1-methyl-6-phenylimidazo [4,5-*b*] pyridine (PhIP) was the HCA with the highest level of consumption in recent studies carried out by our team in a sample of adults from Asturias (northern Spain)^1,3^ due to its wide presence in many types of meat.^4^ PhIP has been classified by the International Agency for Research on Cancer (IARC) as “possibly carcinogenic to humans”^5^ and has been extensively studied in animal models of colorectal cancer (CRC) in combination with the compound sodium dextran sulfate (DSS), which increases the susceptibility to PhIP-induced carcinogenesis of the large bowel.^6,7^ Within the proposed mechanisms for this association, PhIP contributes to the formation of DNA adducts^6^ and preneoplastic lesions as aberrant crypt foci (ACF) in the colonic mucosa^8^ and to a higher incidence and multiplicity of intestinal mucosal lesions and adenocarcinomas in comparison to other HCAs.^9^ However, most studies have used pharmacological doses rather than amounts that could be provided by the usual diet, which range from 80 to 190 ng/d.^6,10^

While evidence is still limited, the consumption of PhIP led to an enrichment of *Lactobacillus* and a reduction of *Prevotellaceae* UCG-001 and *Clostridiaceae* in the gut microbiota of mice.^10^ In addition, while specific gut microbes such as *Bacteroides fragilis*, *Escherichia coli*, and *Enterococcus faecalis* have been linked to CRC,^11−15^ several probiotic strains, including members of the species *Bifidobacterium longum*,^16^*Lactobacillus acidophilus*,^17^ and *Lacticaseibacillus rhamnosus*,^18^ have shown beneficial effects in various murine models of colon cancer. Although the protective mechanisms are unknown, *L. rhamnosus* strains have been specifically associated with induction of epithelial cell apoptosis, and suppression of the nuclear factor kappa B (NFκB) signaling pathway associated with inflammation.^18^

Rebalancing the gut microbiome, enhancing the intestinal mucosal barrier function, and modulating the immune response are among the potential mechanisms that contribute to explaining the beneficial actions of probiotic mixtures.^19^ Also, to act as a carcinogen, PhIP needs to be enzymatically activated in the body, and the colon microbiota has shown to mediate its activation.^20^ PhIP has been extensively studied in animal models in combination with the colitis-promoting compound DSS.^7^ Dietary fiber can act as a sequestering agent for some toxic compounds, contributing to decreasing the intestinal toxicity,^21^ and to modulate the microbiota by promoting the growth of beneficial bacteria and inhibiting some pathogenic groups.^22^ Dietary fiber has evidenced an amelioration of colitis symptoms,^23^ with higher intakes being associated with a lower risk of CRC.^24^ In addition to maintaining gut homeostasis, the production of metabolites such as short-chain fatty acids (SCFA) as end microbial fermentation products of dietary fiber in the intestine may underlie its protective effect.^25^ There is strong evidence suggesting the modulation of gut microbiota by diet and dietary compounds, so further research was performed to elucidate the possible role of HCAs. Previous results pointed to PhIP and dietary-derived bioactive compounds, such as fibers, as potential drivers of gut microbiota.^26^ In addition, significant associations were found between the level of intake of these compounds and shifts of microbiota composition according to the severity of the damage to the colon mucosa.^27^ Based on this evidence, the aim of the present study was to mimic the human context to explore the potential of fiber and probiotics to counteract the impact of the intake of PhIP + DSS on mucosal damage and fecal microbiota in a rat model. For this purpose, we have used a PhIP dose extrapolated from daily dietary intake in humans and DSS to promote inflammation of the intestinal mucosa similar to that produced by long-term consumption of a pro-inflammatory diet.

## Materials and Methods

This animal intervention has been carried out as a part of the broader project “Effect of diet and exposure to xenobiotics generated in food processing on the genotoxic/cytotoxic capacity of the intestinal microbiota” (reference: RTI2018–098 288–B-I00), financed by the Spanish Agency of Research (AEI), that aimed to analyze the impact of the intake of HCAs over damage of colon mucosa and the possible counteracting effect of the intake of probiotics and prebiotics.

### Chemicals

PhIP (Catalogue number: 105650–23–5; Lot number: 134288) was obtained from MedChemExpress (Sollentuna, Sweden) and DSS (M.W. 36–50 kDa; Catalogue number: 160110; Lot number: S7980) was obtained from MP Biomedicals (Loughborough, UK). All animal diets: standard (Standard Rodent Diet A40) and supplemented with probiotics (Standard Rodent Diet A04+MP36) and fiber (Standard Rodent Diet A05) were obtained from SAFE (Augy, France). The probiotic Lactiplus VSL#3 was obtained from PiLeJe (Paris, France). QIAamp Fast DNA Stool Mini Kits were purchased from Qiagen (Sussex, UK).

### Animals

The animal experiment conducted was performed in accordance with the protocols and procedures approved by the Ethics and Animal Experimentation Committee at the University of Oviedo, Spain (PROAE 48/2019). 44 male Fischer 344/NHsd rats (200 g of body weight; 7 weeks old) were purchased from Charles River Laboratories (Les Oncins, France) and maintained at the Bioterium of the University of Oviedo (N° REGISTER: ES 33044 0003591) under controlled 12 h light–dark cycle and at a constant temperature of 22 ± 2 °C and relative humidity of 55 ± 15%. Animals were randomly placed in polypropylene cages (2 to 3 rats per cage) and acclimatized for 1 week with free access to tap water and pelleted food. After acclimatization, each cage was randomly assigned to one of the four groups. Considering the sample size in each group (*n* = 11) and microbial relative abundances in rat feces, the statistical power of our results with a type I error probability of 0.05 is 95–98% (G*Power version 3.1.9.6 Franz Faul, Universität Kiel, Germany).

### Treatments and Experimental Design

**Figure 1:**
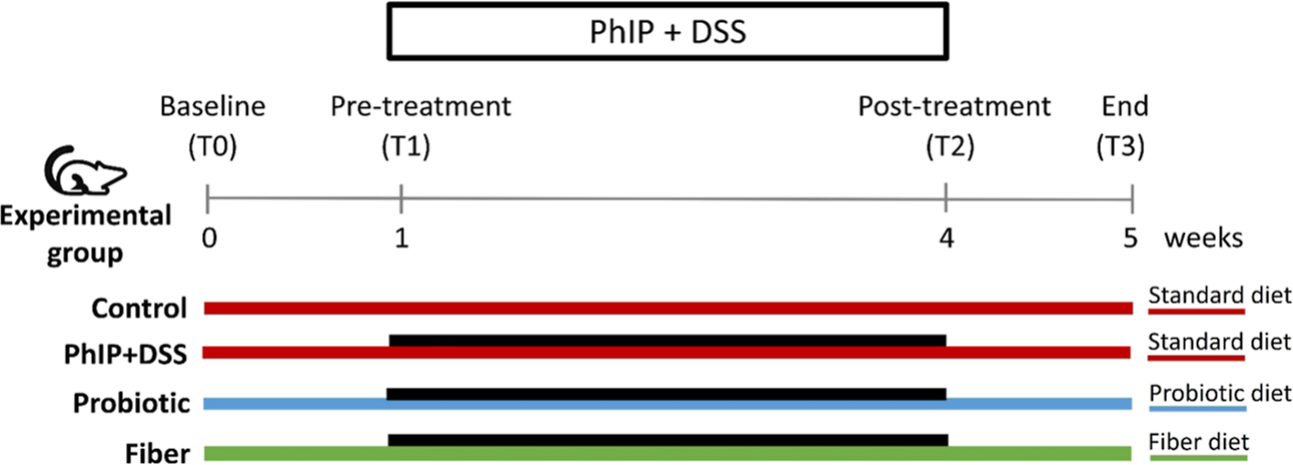
Experimental design. Different colored lines indicate the period of diet administration, and the black line indicates the period of PhIP and DSS administration. Fecal samples were collected at each time interval (T0, T1, T2, and T3). DSS, sodium dextran sulfate, and PhIP, 2-amino-1-methyl-6-phenylimidazo [4,5-*b*] pyridine.

All animals were fed a standard diet during acclimatization, and those in the control and the PhIP + DSS groups were maintained on the standard diet for the entire duration of the intervention (5 weeks). The probiotic group received a customized diet with the probiotic Lactiplus VSL#3 composed of four *Lactobacillus* strains (*L. acidophilusBA05*, *Lactobacillus plantarumBP06*, *Lactobacillus paracaseiBP07*, and *Lactobacillus helveticusBD08z*), three *Bifidobacterium* strains (*Bifidobacterium breveBB02*, *Bifidobacterium animalis* subsp. *lactis BL03x*, and *B. animalis* subsp. *lactis BL04y*), and one *Streptococcus* strain (*Streptococcus thermophilusBT01*). The probiotic was incorporated into the diet as a lyophilized powder and consumed by animals at 2.2 × 10^9^ CFU/d. The fiber group received a prebiotic diet containing 5.9% (w/w) of fiber. All customized diets were provided by the company SAFE (Augy, France) in pellet form and were available *ad libitum*. Their compositions are detailed in Table 1. Probiotic and fiber diets were administered from baseline (T0) to the end of the study (T3) (5 weeks), as described in Figure 1.

**Table 1:** Nutritional Composition of the Commercial Diets Administered in the Study

	standard^c^	probiotic^d^	fiber^e^
energy content (kcal/g)^a^	3.34	3.21	3.22
*macronutrients*			
carbohydrates^b^			
g/kg	610	580	617
kcal (% of total)	73.05	72.27	76.65
protein			
g/kg	152	154	118
kcal (% of total)	18.20	19.19	14.66
fats			
g/kg	32	30	31
kcal (% of total)	8.62	8.33	8.66
*supplementation*			
probiotic (mg/kg)	0	1300	0
fiber (%)	4.10	3.74	5.90

a Metabolizable energy in the standard, probiotic, and fiber diet was 3.10, 3.15, and 2.83 kcal/g, in each case.

b Nitrogen-free extract representing sugars and starches.

c Standard Rodent Diet A40, SAFE, Augy, France.

d Standard Rodent Diet A04+MP36, SAFE, Augy, France.

e Standard Rodent Diet A05, SAFE, Augy, France.

The experimental groups (*n* = 11 animals/group) were the negative control (standard diet), the positive control, or PhIP + DSS (standard diet + PhIP + DSS), and the intervention groups: probiotic (probiotic diet + PhIP + DSS) and fiber (fiber-enriched diet + PhIP + DSS) (Figure 1). After 1 week of customized diet supplementation (T1), animals in the PhIP + DSS, probiotic, and fiber groups were administered the dietary carcinogen PhIP and DSS. PhIP was administered after anesthesia with isoflurane by oral gavage at 1 mg/kg of body weight (1 ppm) in sterile water. DSS (1.5%, w/v) was dissolved in tap water at room temperature and available *ad libitum* in substitution of normal drinking water. Animals in the PhIP + DSS, probiotic, and fiber groups were exposed to 3 weeks of PhIP + DSS treatment (5 days of treatment per week) (Figure 1). The control group was anesthetized for the administration of sterile water by oral gavage in substitution of PhIP. PhIP was effective in inducing carcinogenesis in previous studies combined with 1.5% DSS,^6^ and the selected concentration of PhIP replicates reported human consumption levels in a previous study (188 ng/day).^1^ After PhIP + DSS administration (T2), rats underwent 1 week of washout without PhIP + DSS and were sacrificed by CO_2_ asphyxiation (T3). All animals survived the intervention (n = 44). Cecum and colon were then excised. Body weight and water and food intake were recorded daily throughout the study, and mean values obtained are presented in Table S1.

### Colon Length and Histologic Assessment

Colon length was measured in a relaxed position without stretching from the ileocecal junction by using a 1 cm square grid. This information is available for only 21 animals. After measurement, feces were removed, and the colon was flushed with phosphate-buffered saline (PBS) to remove residual bowel content and divided into proximal, mild, and distal colon. The 3 segments from a total of 44 rats were then stored in formaldehyde. Swiss rolls were prepared by fixing each colon segment in 10% formaldehyde, embedding them in paraffin, and then sectioning into 3 μm thick slices for hematoxylin–eosin staining. A total of 528 histological sections (4 sections per colon segment within each animal) were examined with NanoZoomer S20MD (Hamamatsu Photonics, France) for the presence of the following features: erosion, crypt loss, diffuse, and focal inflammation, and ACF without cell alterations, hyperplasia, or dysplasia. The results obtained are presented as the proportion of colon segments affected by each or all histologic features. No ACF was observed in the samples of study.

### Feces Collection, DNA Extraction, Analysis of Fecal Microbiota Using High Throughput Sequencing, and SCFA Determinations

Fecal samples were collected in sterile containers at baseline (T0), before PhIP + DSS administration or pretreatment (T1), after PhIP + DSS administration or post-treatment (T2), and at the end of the study (T3) (Figure 1). Fecal samples were obtained in the morning, directly from each animal, to avoid contamination. However, at given intervals, some animals did not excrete fresh feces at any of the intervals of study (*n* = 8). Samples were stored at −80 °C for further analyses. The stool microbiota composition was determined by 16S rRNA gene sequencing for 36 animals. Fecal samples were diluted 1/7 (w/v) in sterile PBS solution and homogenized for 3 min at full speed in a LabBlender 400 stomacher (Seward Medical, London, UK). They were centrifuged for 15 min at 4 °C and 14,000 rpm, and the supernatants obtained were separated from the pellets and kept frozen at −20 °C until use. DNA was extracted by using the QIAamp Fast DNA Stool Mini Kit with an additional bead-beating step. The quantification of DNA and the determination of the 260/280 ratio were performed using the Take3Micro-Volume plate and Gen5 microplate reader (BioTek Instrument Inc., Winooski, VT, USA). The DNA obtained was kept frozen at −80 °C until analysis. Variable region V3–V4 of bacterial 16S rRNA genes present in each fecal community was amplified by PCR, and the resulting amplicons were sequenced on an Illumina NovaSeq 6000 platform instrument. The obtained individual sequence reads were filtered to remove low-quality sequences. All Illumina quality-approved, trimmed, and filtered data were integrated to generate de novo 16S rRNA Amplicon Sequence Variants with ≥97% sequence homology using Uparse software (Uparse v7.0.1090). A classification of all reads to the lowest possible taxonomic rank was performed using Quantitative Insights Into Microbial Ecology (QIIME2) and a reference data set from the SILVA 138 database. The 121 fecal samples analyzed yielded an average of ∼90,000 filtered partial sequences per sample and a final number of 3826 total ASVs. The whole procedure of sequencing and annotation was undergone at Novogene Bioinformatics Technology Co., Ltd. For the representation of the obtained results, only taxa with relative abundance greater than 1% in at least two samples and obtained mean values were considered. SCFA were analyzed by gas chromatography from the supernatants of 1 mL of the homogenized feces.^28^ A chromatograph 6890N (Agilent Technologies Inc., Palo Alto, CA, USA) connected to a mass spectrometry detector 5973N (Agilent Technologies) and a flame ionization detector was used for the identification and quantification of SCFA, as described in previous works.^29^

### Statistical Analysis

Results were analyzed using IBM SPSS software version 27.0 (IBM SPSS, Inc., Chicago, IL, USA). The goodness of fit to the normal distribution was checked by means of the Kolmogorov–Smirnov test. The analysis of categorical variables was performed with the *X*^2^ test. Continuous variables were analyzed through analysis of the variance (ANOVA) and Tukey’s posthoc HSD tests for intragroup comparisons to detect differences along the study within the same group and through *T*-test for pairwise comparisons at a specific time interval to detect differences between the control vs PhIP + DSS group, the PhIP + DSS vs probiotic group, and the PhIP + DSS vs fiber group. Spearman correlation analyses were conducted, and heatmaps were generated using the ClustVis web tool.^30^ Graphical representations were obtained using GraphPad Prism 8 (La Jolla, CA, USA), and the Table of Contents was created using BioRender software.

## Results

### Food Intake and Body Weights

No differences in body weight between the four groups were found at the beginning or end of intervention, but the fiber group presented a lower body weight gain in comparison to the PhIP + DSS group (54 vs 69 g; *p* = 0.003) (Table S1), probably due to the lower metabolizable energy (2.83 vs 3.44 kcal/g, respectively) (Table 1). The daily consumption of food and water was monitored daily, and no significant differences were found between groups.

### Impact of PhIP + DSS on Colon Length and Mucosal Damage and the Counteracting Effect by Probiotic or Fiber Supplementation

**Figure 2:**
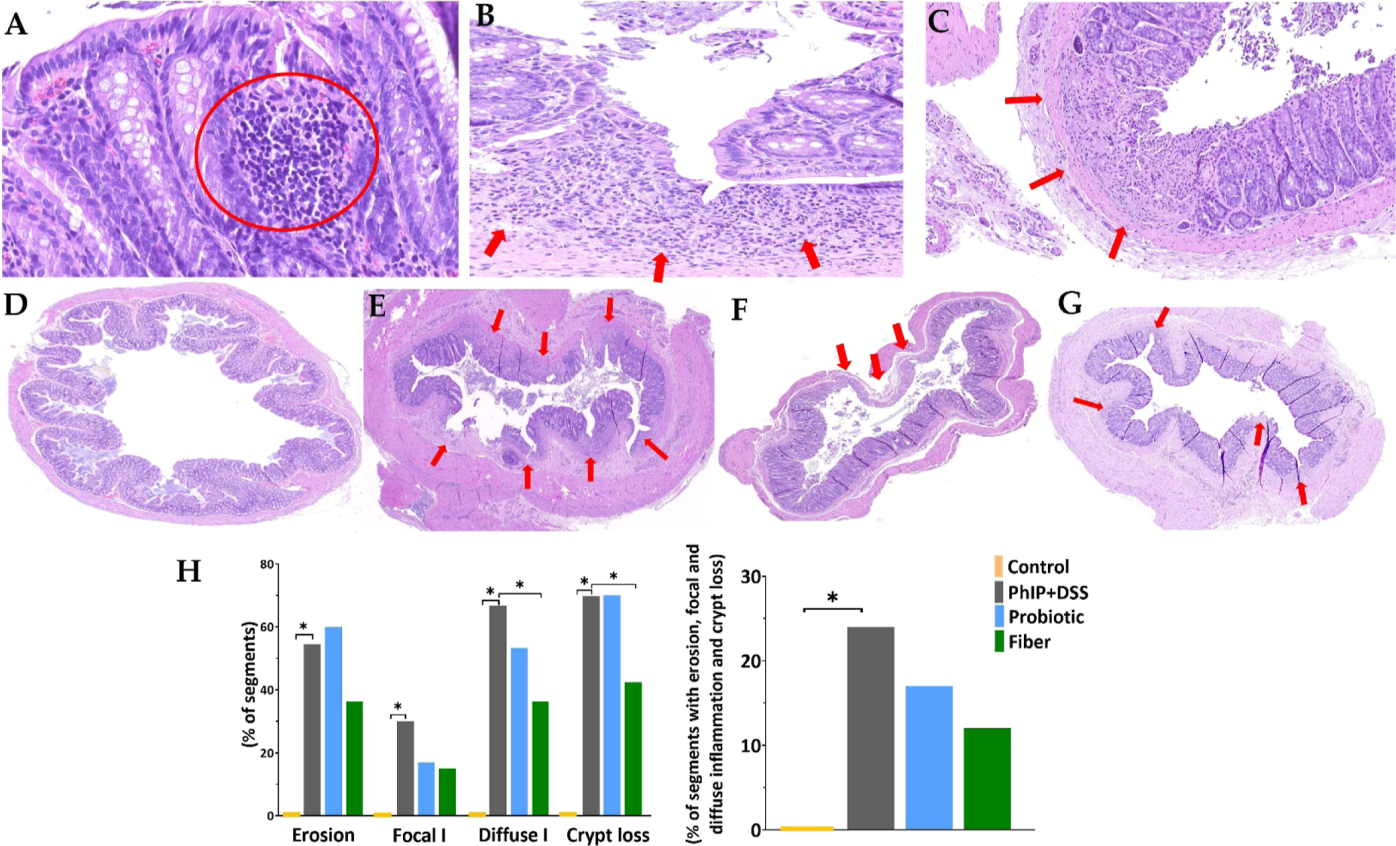
Histologic colonic mucosal damage induced by PhIP + DSS treatment and counteracting effect by probiotic or fiber supplementation. Histologic sections stained with hematoxylin–eosin showing (A) focal I through lymphoid infiltration, ×30; (B) diffuse I, ×20; and (C) erosion and crypt loss, ×10. Histologic sections of distal colon of (D) control group ×2; (E) PhIP + DSS group ×2.5; (F) probiotic group ×2.5; and (G) fiber group ×2.5. Red arrows indicate erosion, diffuse I, and crypt loss. (H) Percentage of colon segments presenting each histologic feature: erosion, focal and diffuse I, and crypt loss; and percentage of colon segments presenting all features within each group. Statistical analysis for (H) pairwise comparisons of the control vs PhIP + DSS group, the PhIP + DSS vs probiotic group, and the PhIP + DSS vs fiber group was performed with the *X*^2^ test (*n* = 33 for each group) (*p < 0.05). DSS, sodium dextran sulfate; I, inflammation; PhIP, 2-amino-1-methyl-6-phenylimidazo [4,5-*b*] pyridine.

The administration of 1 ppm of PhIP and 1.5% (w/v) of DSS for 3 weeks damaged the colonic mucosa of rats in the PhIP + DSS group (Figure 2). The histologic features assessed by hematoxylin–eosin stain are depicted in Figure 2A–C and include focal (Figure 2A) and diffuse (Figure 2B) inflammation and erosion and crypt loss (Figure 2C). In Figure 2D, the colonic mucosa of the control group receiving a standard diet is presented without lesions. In comparison to controls, the PhIP + DSS treatment group (Figure 2E) presented colon segments with erosion (55 vs 0%; *p* < 0.001), crypts loss (70 vs 0%; *p* < 0.001), focal (30 vs 0%; *p* < 0.001), and diffuse (67 vs 0%; *p* < 0.001) inflammation (Figure 2H). Similar results were obtained for the proportion of colon segments presenting all histologic findings simultaneously in the PhIP + DSS treatment group vs controls (24 vs 0%; *p* = 0.003) (Figure 2H). No ACF was found in this work. Considering the three colonic segments, the distal colon presented a higher grade of histologic alteration (Figure S1). In the PhIP + DSS group, crypt loss and diffuse inflammation were present in 91% of the distal segments and in 45% of the proximal segments, while focal inflammation was more common in proximal segments (64%) compared to mild (9%) and distal (18%) segments (Figure S1).

The administration of 5.9% (w/w) fiber counterbalanced colonic mucosal damage (Figure 2G). In comparison to the PhIP + DSS group, the fiber group presented a reduced proportion of segments with diffuse inflammation (36 vs 67%; *p* = 0.014) and crypts loss (42 vs 70%; *p* = 0.026) (Figure 2H). The administration of the probiotic (Figure 2F) evidenced a similar tendency toward alleviation of histologic damage compared to the PhIP + DSS group through nonstatistically significant reductions in the proportion of segments with focal (17 vs 30%; *p* = 0.204) and diffuse inflammation (53 vs 67%; *p* = 0.280) (Figure 2H).

**Figure 3:**
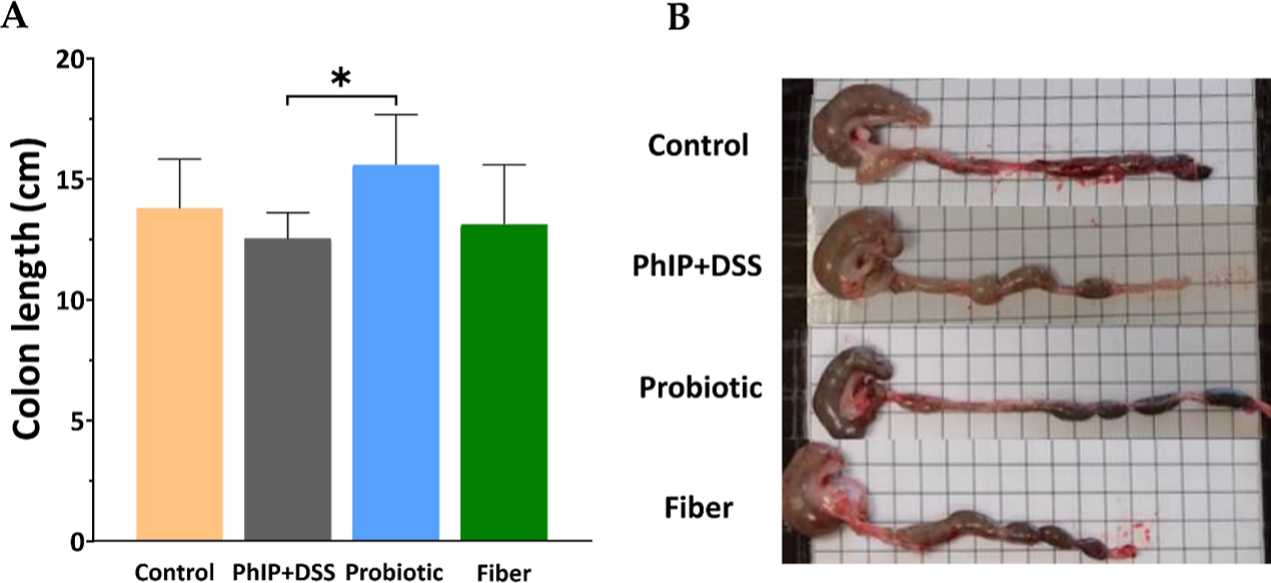
Impact of PhIP + DSS on colon length and counteracting effect by probiotic or fiber supplementation. (A) Colon length of each group. Bar plots represent the mean ± SD values obtained. Statistical analysis for pairwise comparisons of the control vs PhIP + DSS group, the PhIP + DSS vs probiotic group, and the PhIP + DSS vs fiber group was performed through a *T*-test (Control *n* = 6, PhIP + DSS *n* = 5, Probiotic *n* = 5, Fiber *n* = 5) (*p < 0.05). (B) Macroscopic appearance of the colon after excision. PhIP, 2-amino-1-methyl-6-phenylimidazo [4,5-*b*] pyridine.

In addition to histologic assessment, the colon length was measured (Figure 3A–B). Whereas a nonsignificant 10% reduction in colon length was found in the PhIP + DSS group compared to controls (12.54 vs 13.80 cm; *p* = 0.695), the colon was 24% longer in the probiotic group when compared to the PhIP + DSS treatment group (15.60 vs 12.54 cm; *p* = 0.019). No significant differences in colon length were found between PhIP + DSS and fiber groups.

### Impact of PhIP + DSS Treatment on Gut Microbiota Composition and Counteracting Effect by Probiotic or Fiber Supplementation

**Figure 4:**
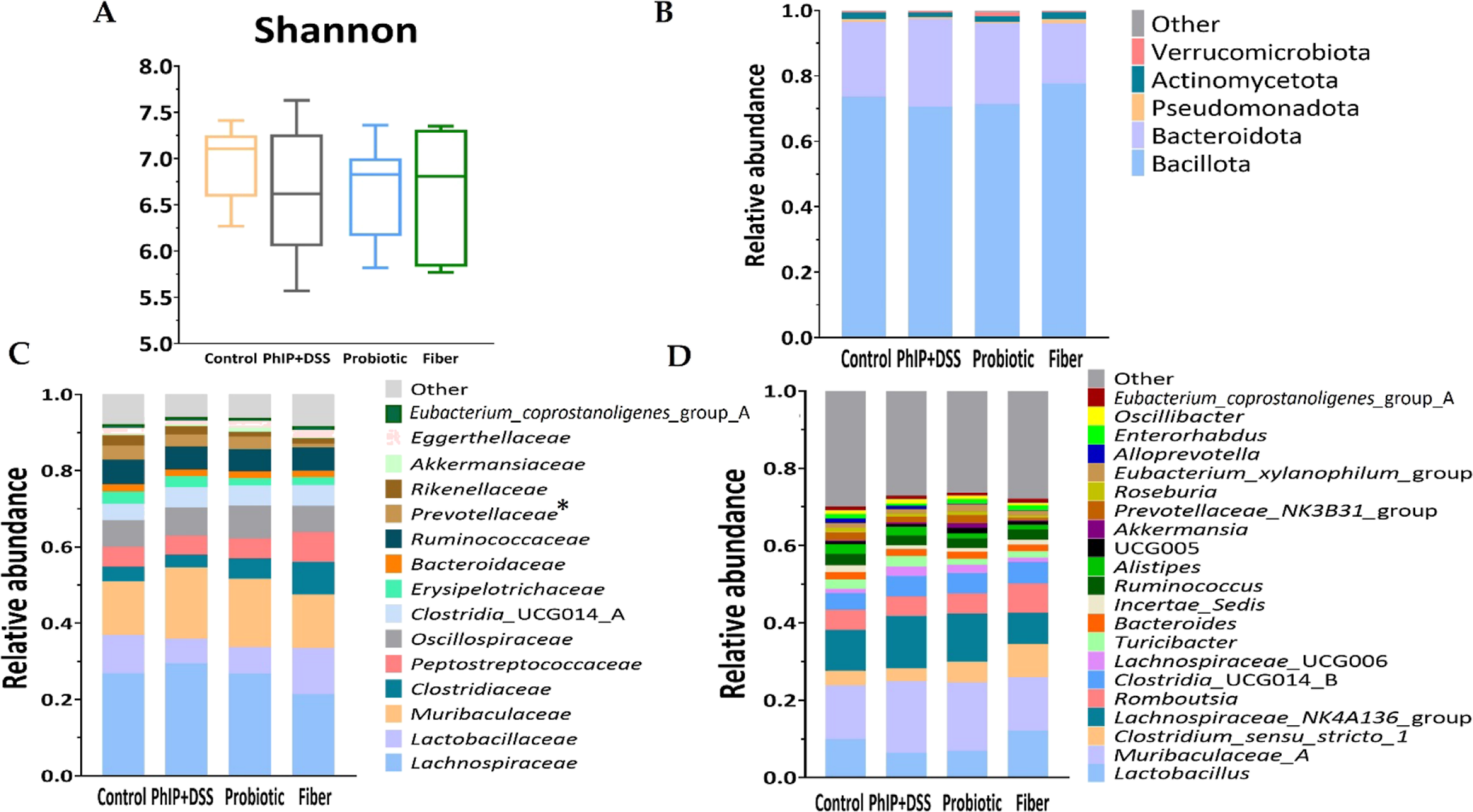
Baseline diversity and profile composition of the gut microbiota in each group. (A) Shannon diversity index. The lines within the boxes represent the median, and the bounds of boxes represent the first and third quartiles (25th and 75th percentiles, respectively). The whiskers denote the lowest and highest values within 1.5 times the IQR from the first and third quartiles, respectively. Microbiota profile composition at (B) phylum, (C) family, and (D) genus level. Bar plots represent the mean values obtained for each taxa. Only those with a mean relative abundance greater than 1% are shown. Statistical analysis for (A–D) pairwise comparisons of the control vs PhIP + DSS group, the PhIP + DSS vs probiotic group, and the PhIP + DSS vs fiber group at the baseline was performed through a *T*-test, and results were found only for the PhIP + DSS vs fiber groups (Control *n* = 8, PhIP + DSS *n* = 7, Probiotic *n* = 8, Fiber *n* = 7) (*p < 0.05). PhIP, 2-amino-1-methyl-6-phenylimidazo [4,5-*b*] pyridine.

The metataxonomic analyses based on sequencing the V3–V4 region of the 16S rRNA gene revealed substantial differences in the composition of the fecal microbiota between the different groups of treatment. Globally, the microbial communities of all samples from the four groups of animals were assigned to 5 phyla, 28 families, and 52 genera. At baseline, no differences were found in alpha diversity for the Shannon index (Figure 4A) and in microbiota composition at the taxonomic levels of phylum, family, and genus among the four experimental groups (Figure 4B–D), except for the family *Prevotellaceae*, which presented a reduced relative abundance in the fiber group compared to the PhIP + DSS group (1.0 vs 3.1%; *p* = 0.027). At the phylum level, Bacillota was the most abundant, followed by Bacteroidota and Actinomycetota. At the family level, the most abundant was *Lachnospiraceae*, followed by *Muribaculaceae*, *Oscillospiraceae* and *Lactobacillaceae*.

### Longitudinal Analysis

**Figure 5:**
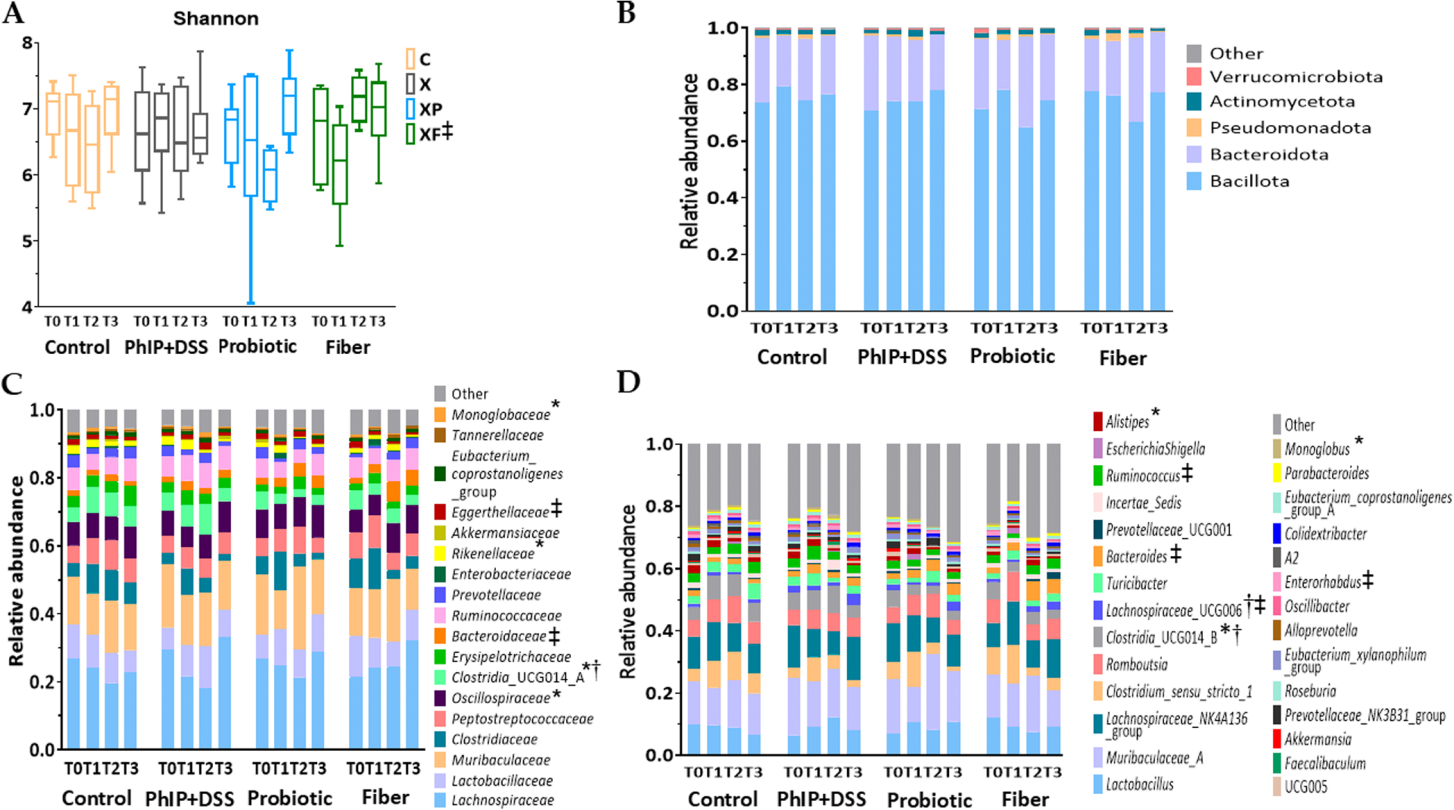
Gut microbiota diversity and profile composition across the study for each group. (A) Shannon diversity index. The lines within the boxes represent the median, and the bounds of boxes represent the first and third quartiles (25th and 75th percentiles, respectively). The whiskers denote the lowest and highest values within 1.5 times the IQR from the first and third quartiles, respectively. Microbiota profile composition at (B) phylum, (C) family, and (D) genus level. Bar plots represent the mean values obtained for each taxa, and only those with a mean relative abundance greater than 1% are shown. Statistical analysis (A–D) for differences across the study (T0, T1, T2, and T3) was performed by one-way ANOVA for each group (Control *n* = 8, *n* = 8, *n* = 9, and *n* = 9; PhIP + DSS *n* = 7, *n* = 8, *n* = 5, and *n* = 9; Probiotic *n* = 8, *n* = 7, *n* = 4, and *n* = 7; and Fiber *n* = 7, *n* = 8, *n* = 8, and *n* = 9 at T0, T1, T2, and T3, respectively) (*p* < 0.05 for PhIP + DSS (*), Probiotic (†), and Fiber (‡) groups). No significant differences were found in the control group across the study. p-values are provided in Tables S2–S5. PhIP, 2-amino-1-methyl-6-phenylimidazo [4,5-*b*] pyridine; T0, baseline; T1, pretreatment; T2, post-treatment; and T3, end of the study.

The longitudinal effect of the intervention on the Shannon index and microbiota composition within each group of animals is shown in Figure 5A–D and Tables S2–S5. Significant variations in the Shannon diversity index along the intervention were detected only in the fiber group, for which the highest diversity index was obtained after PhIP + DSS administration (T2) (Figure 5A).

Regarding the gut microbiota composition, the PhIP + DSS group of treatment presented variations along the intervention in the relative abundance of the family *Clostridia* UCG014_A and the genus *Clostridia* UCG014_B, the families *Oscillospiraceae* and *Monoglobaceae* and the genus *Monoglobus*, and the family *Rikenellaceae* and the genus *Alistipes* (Figure 5B–D). Specifically, in the case of *Monoglobaceae* and the genus *Monoglobus* as well as for *Clostridia* UCG014 (family and genus) (Figure S2), the PhIP + DSS treatment (T2) promoted the increase of these microbial groups, followed by a significant reduction in their relative abundance 1 week after the cessation of PhIP + DSS administration (T3) from 1.2 to 0.4% (*p* = 0.040) (*Monoglobaceae* and *Monoglobus*) and from 9.0 to 3.9% (*p* = 0.013) (*Clostridia* UCG014, family and genus). These results are in contrast to the increased relative abundance of *Oscillospiraceae* from pretreatment (T1) to 1 week after cessation of the administration of PhIP + DSS (T3) (from 6.3 to 9.3%; *p* = 0.031) in the same group. In Figure 5B–D, it can be observed that the probiotic group also presented variations along the study in the relative abundances of *Clostridia* UCG014 (at the family and genus level) and *Lachnospiraceae* UCG006. Among them, an increment in the relative abundance of *Lachnospiraceae* UCG006 was detected from pretreatment (T1) to 1 week after cessation of PhIP + DSS administration (T3), (from 0.8 to 3.0%; *p* = 0.035) (Figure S2), and the opposite nonsignificant trend was observed for the relative abundance of *Clostridia* UCG014 (family and genus) in comparison to the PhIP + DSS group: from 5.3% at baseline (T0) to 2.5% after PhIP + DSS administration (T2) (*p* = 0.088) (Figure S2). The fiber group presented variations throughout the intervention in the relative abundance of the phylum Actinomycetota and related taxa *Eggerthellaceae* and *Enterorhabdus*, as well as *Bacteroidaceae* and *Bacteroides*, and genera *Lachsnopiraceae* UCG006 and *Ruminococcus* (Figure 5B–D). Whereas in comparison to baseline (T0) reduced relative abundance at the end of the study (T3) was noted in the case of Actinomycetota (from 2.1 to 0.7%; *p* = 0.016), *Eggerthellaceae* (from 1.9 to 0.5%; *p* = 0.008), and *Enterorhabdus* (from 1.2 to 0.4%; *p* = 0.009), an increasing trend from pretreatment (T1) was presented by *Ruminoccus* (from 1.3 to 3.2%; *p* = 0.033) (Figure S2).

### Cross-Sectional Analysis

**Figure 6:**
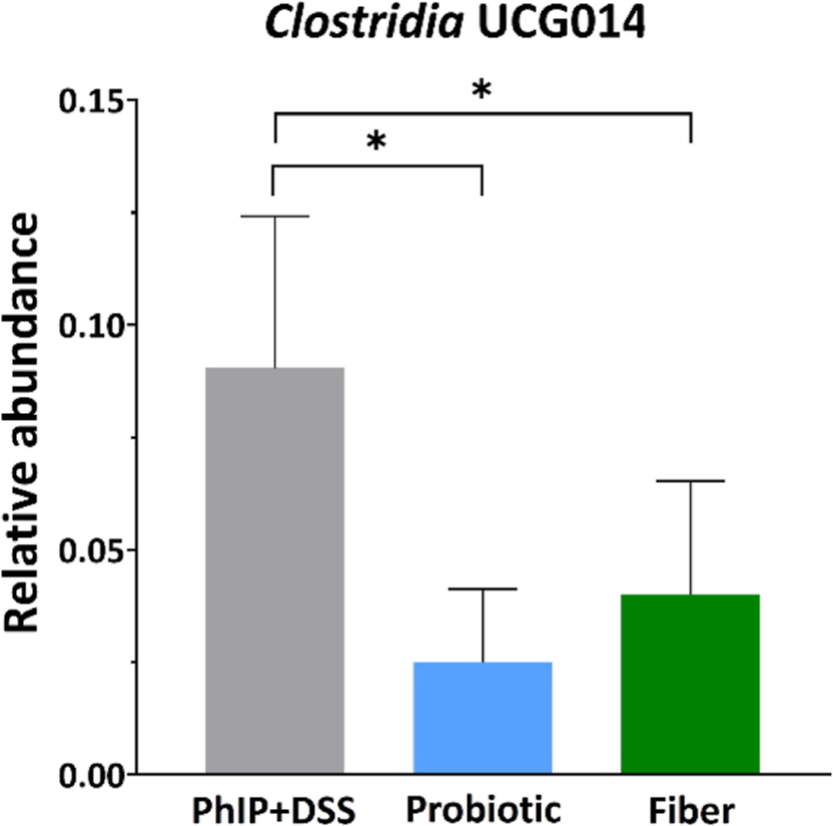
Effect of probiotic or fiber supplementation on the relative abundance of *Clostridia* UCG014 after PhIP + DSS administration. Bar plots represent mean relative abundance ±SD. Statistical analysis for pairwise comparisons of the PhIP + DSS vs probiotic group and the PhIP + DSS vs fiber group at post-treatment was performed by a *T*-test (PhIP + DSS *n* = 5, Probiotic *n* = 4, Fiber *n* = 8) (*p < 0.05). Only microbial groups with significant differences for both comparisons are shown. DSS, sodium dextran sulfate; PhIP, 2-amino-1-methyl-6-phenylimidazo [4,5-*b*] pyridine.

The effect of the different diets was determined at the end of the intervention. Compared to the PhIP + DSS group, both the probiotic and fiber supplementations reduced the relative abundance of *Clostridia* UCG014 (family and genus) (2.5 and 4.0% vs 9.0%; *p* = 0.010, both cases) (Figure 6). In comparison to the PhIP + DSS group, supplementation with the probiotic led to reduced relative abundance of *Eubacterium coprostanoligenes* group (family and genus) (0.4 vs 1.2%; *p* = 0.032) and *Ruminococcus* (1.8 vs 2.7%; *p* = 0.028) (Figure S3), whereas supplementation with fiber reduced the relative abundance of *Monoglobaceae* and *Monoglobus* (0.4 vs 1.2%; *p* = 0.021) and UCG005 (0.6 vs 1.2%: *p* = 0.030) and increased *Bacteroidaceae* and *Bacteroides* (6.1 vs 1.9%; *p* = 0.040) and *Alistipes* (1.6 vs. 0.7%; *p* = 0.041) (Figure S3).

**Figure 7:**
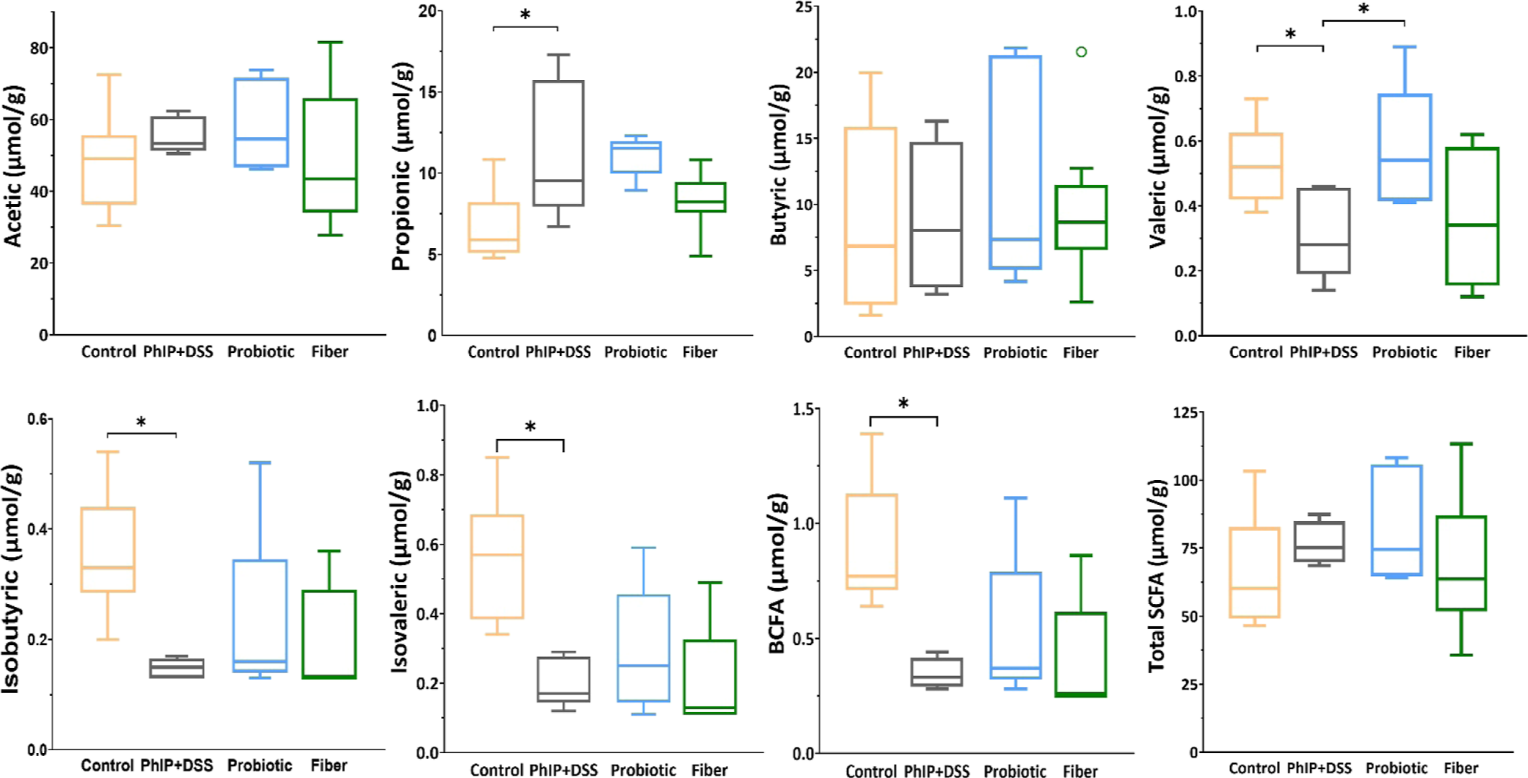
Analysis of fecal SCFA after the administration of PhIP + DSS. The lines within the boxes represent the median, and the bounds of the boxes represent the first and third quartiles (25th and 75th percentiles, respectively). The whiskers denote the lowest and highest values within 1.5 times the IQR from the first and third quartiles, respectively. Statistical analysis for pairwise comparisons of the control vs PhIP + DSS group, the PhIP + DSS vs probiotic group, and the PhIP + DSS vs fiber group at post-treatment was performed through *T*-test (Control *n* = 9, PhIP + DSS *n* = 5, Probiotic *n* = 4, and Fiber *n* = 8) (*p < 0.05). BCFA, branched chain fatty acids; DSS, sodium dextran sulfate; PhIP, 2-amino-1-methyl-6-phenylimidazo [4,5-*b*] pyridine; and SCFA, short chain fatty acids.

The level of fecal SCFA after the administration of PhIP + DSS for each group of animals is displayed in Figure 7. The PhIP + DSS group showed significantly higher fecal levels of propionic acid (9.53 vs 5.90 μmol/g; *p* = 0.015) and lower levels of isobutyric acid (0.15 vs 0.33 μmol/g; *p* = 0.001), isovaleric acid (0.17 vs 0.57 μmol/g; *p* = 0.001), branched SCFA (BCFA) (0.33 vs 0.77 μmol/g; *p* = 0.001), and valeric acid (0.28 vs 0.52 μmol/g: *p* = 0.014) as compared to controls. The administration of the probiotic led to a partial restoration of valeric acid excretion levels, and increased concentrations were observed in comparison to the PhIP + DSS group (0.54 vs 0.32 μmol/g; *p* = 0.042). No significant differences in fecal SCFA levels were found in the fiber group compared to the PhIP + DSS group after PhIP + DSS administration, according to the *T*-test.

**Figure 8:**
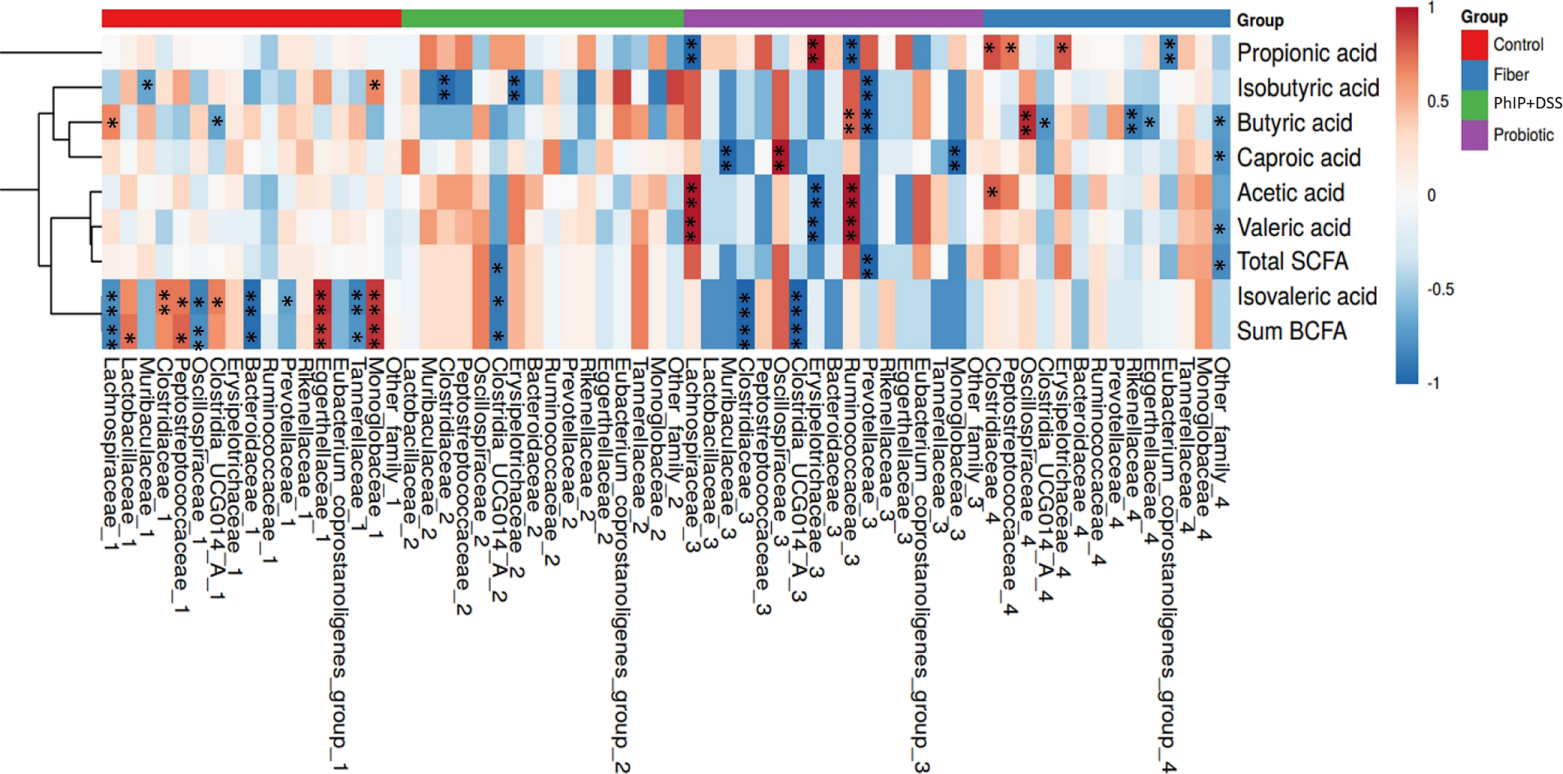
Spearman correlations representation between the level of fecal SCFA (rows) and most abundant bacterial families (columns) by group of study after the administration of PhIP + DSS. (*) and (**) *p* < 0.05 and 0.01, respectively. Only taxa showing mean relative abundances higher than 1% are shown. (Control *n* = 9, PhIP + DSS *n* = 5, Probiotic *n* = 4, and Fiber *n* = 8). BCFA, branched chain fatty acids; DSS, sodium dextran sulfate; PhIP, 2-amino-1-methyl-6-phenylimidazo [4,5-*b*] pyridine; and SCFA, short chain fatty acids.

**Figure 9:**
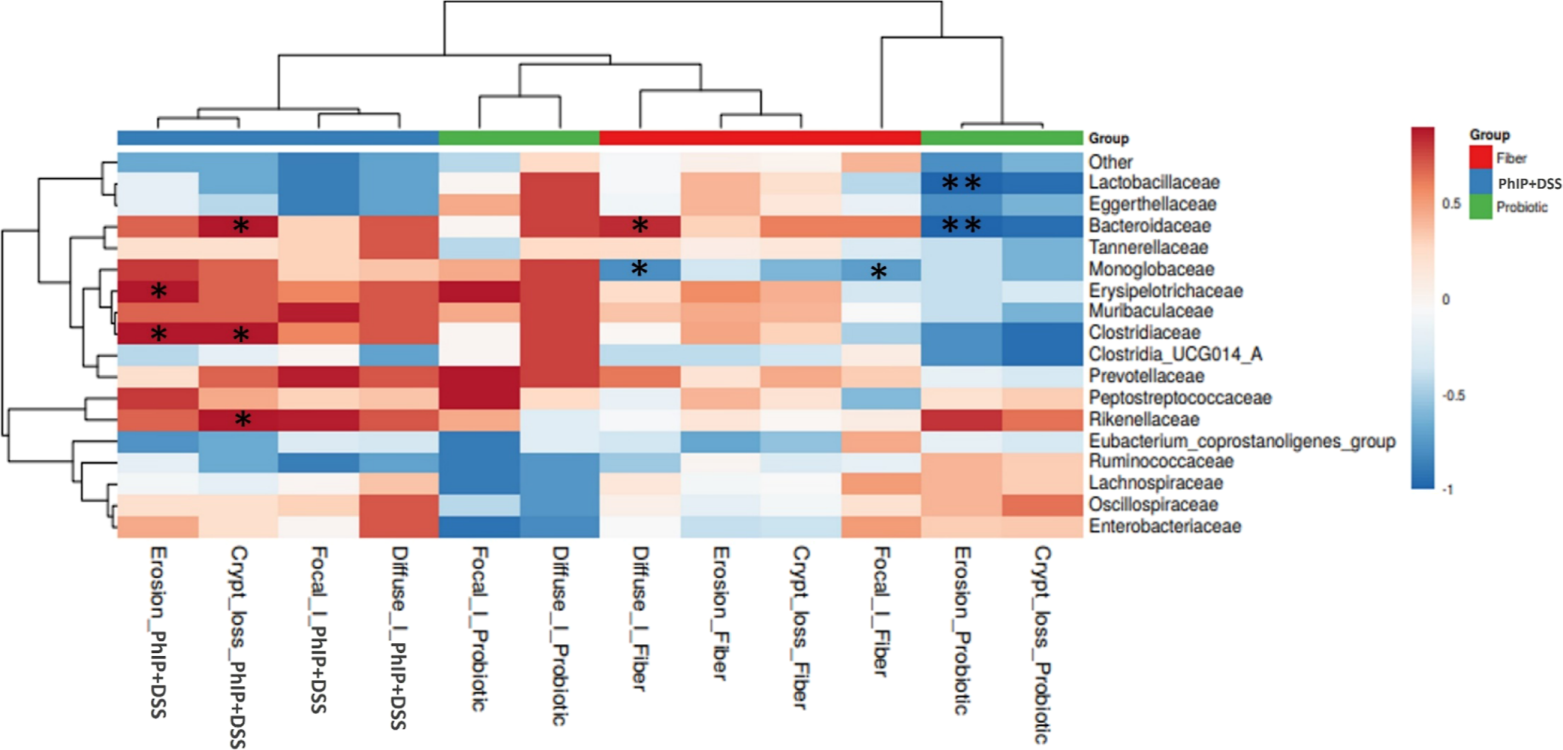
Spearman correlations representation between most abundant bacterial families (rows) and the histological alterations (erosion, diffuse and focal inflammation, and crypt loss) by group of study after the administration of PhIP + DSS. (*), (**) *p* < 0.05 and 0.01, respectively. Only taxa showing mean relative abundances higher than 1% are shown. No histological alterations were found in the control group. (PhIP + DSS *n* = 5, Probiotic *n* = 4, and Fiber *n* = 8). DSS, sodium dextran sulfate and PhIP, 2-amino-1-methyl-6-phenylimidazo [4,5-*b*] pyridine.

The associations found between the relative abundance of gut microbiota families and the concentration of SCFA excreted in feces for each experimental group after PhIP + DSS administration are shown in Figure 8. The control group presented a direct association between *Clostridia* UCG014_A and isovaleric acid, and the opposite direction was found for this association in the case of animals from the PhIP + DSS group of treatment and the probiotic group. The group supplemented with the probiotic presented the greatest number of significant associations and is the only one noting significant associations with valeric acid through direct correlations with the *Ruminococcaceae* and *Lachnospiraceae* families and inversely with the family *Erysipelotrichaceae*. In addition, the relative abundances of gut microbiota families after PhIP + DSS administration were correlated with the occurrence of histological alterations according to the experimental group (Figure 9). The PhIP + DSS group presented direct correlations between the occurrence of erosion and crypt loss and the relative abundance of *Clostridiaceae*, and inverse associations were found in the fiber group between focal and diffuse inflammation and the relative abundance of *Monoglobaceae*. In the probiotic group, inverse associations were found between erosion and the levels of *Bacteroidaceae* and *Lactobacillaceae*.

## Discussion

In this work, an animal PhIP + DSS model was used to mimic the potential of dietary fiber and a mixed probiotic to counteract the impact of the intake of HCAs formed during the cooking of foods such as meat on colon damage and gut microbiota. PhIP has been shown to promote the formation of genotoxic metabolites and DNA adducts over time, and DSS increases PhIP susceptibility of colonic epithelial cells.^31^ The coutilization of PhIP with DSS has been previously shown to shorten the time of progression of PhIP-induced tumors in murine models from 52 to 82 weeks (when PhIP is administered alone) to 6–24 weeks.^6^ In our study, the administration of 1 ppm of PhIP concomitantly with DSS to Fischer 344 rats for 3 weeks resulted in an effective combination to provoke histologic damage in the colonic mucosa through erosion, focal and diffuse inflammation, and crypt loss. This supports the model of short-term induction of carcinogenesis used in the present work as suitable for investigating diet-related colon carcinogenesis^32^ by mimicking the long-term exposure to PhIP. Also, the lower concentrations of PhIP (1 ppm) employed in this work as compared to previous studies were used based on the level of consumption observed among humans in previous works from our team.^1,3^

The greater presence of histologic features in distal segments is similar to the distribution of colon tumors reported in human studies during the early onset of CRC^33^ and is also in accordance with the absence of tumors found after a similar intervention study conducted by other authors (5 day treatment with PhIP at lower doses of administration than the used by us (0.1 mg/kg)).^6^ In contrast to the higher concentrations of PhIP used in some previous studies, aiming to induce a tumor in the minimum time possible,^6,7,10^ the dose administered in the present work is comparable to the estimated levels of the regular consumption of this compound in humans.^34^ Colon length has been widely used as a morphologic marker of the degree of inflammation, as its shortening is associated with relevant histological changes^23^ and carcinogenesis.^35^ The nonsignificant trend toward colon shortening (10%) observed in our experimental model is consistent with these studies, considering that the doses employed were not sufficient to induce carcinogenicity.

There is a consensus regarding the involvement of gut microbiota in the development of inflammation processes in the colon mucosa. The histologic findings in the present study are parallel to the increased relative abundance of *Clostridia* UCG014, and in the case of erosion and crypt loss, these were directly associated with the relative abundance of *Clostridiaceae*. This lies in accordance with the results obtained by other authors, in which higher relative abundances of *Clostridiaceae*_1 and *Clostridium_sensu_stricto*_1 were detected after similar intervention periods and the administration of slightly higher doses of PhIP (10 ppm).^10^ The family *Clostridiaceae* has already been identified as pro-inflammatory,^36^ and the levels of *Clostridia* UCG014 are elevated in colitis^23^ and CRC^37^ models. The increase of Clostridia in the group of animals treated with PhIP + DSS was parallel to a greater excretion of propionic acid and reduced valeric and BCFA, as previously reported in a chemically induced CRC animal model (azoxymethane (AOM)/DSS).^36^ Among these, inverse associations were found between *Clostridia* UCG014_A and BCFA in this work, which have previously been associated with the impairment of gut barrier integrity.^38^

To our knowledge, this is the first study analyzing the effect of the administration of a mixed probiotic on the gut microbiota composition to reduce the negative impact of dietary xenobiotics at the intestinal level. There is strong evidence suggesting the convenience of using a combination of probiotics rather than a single strain in the modulation of gut microbiota.^19^ In this sense, the intake of the multistrain probiotic composed of *Lactobacillus*, *Bifidobacterium*, and *Streptococcus* strains in this work (2.2 × 10^9^ CFU/d) were based on the recommended intake in humans (10^9^ CFU/d), considering an adult with an average body weight of 70 kg, and in consonance with our results, it has shown beneficial effects in colitis and in the progression of colonic neoplastic lesions when used at doses around 2 × 10^9^ CFU/d in previous animal studies.^39^ It was also found in the present work that probiotic administration counteracted colon shortening in comparison to that of the PhIP + DSS group. These results are in consonance with the longer colon length reported by other authors after supplementation with the same combination of probiotics in experimental colitis models.^40^ In addition, the previously reported increased colon length was not associated with variations in leukocyte infiltration in the lamina propria and submucosa.^41^ This may indicate a colon elongation effect by probiotic supplementation independent of inflammation of the colonic mucosa, which would explain the nonsignificant variations in the proportions of colon segments with histological features observed after the probiotic supplementation in this work. In addition, erosion was found to be inversely associated with *Lactobacillaceae* and *Bacteroidaceae* in this group. In the present work, we have found that the longer colon length was parallel to an increased abundance of *Adlercreutzia* compared to the PhIP + DSS group (0.7 vs 0.3%, *p* = 0.010, data not shown); this microorganism was previously positively associated with colon length,^42^ amelioration of colitis,^43^ and excreted valeric acid.^44^ The probiotic promoted an increase in the fecal concentration of SCFA valeric acid, restoring the levels obtained in the control group. This SCFA has been directly correlated with the circulating levels of anti-inflammatory cytokines in previous works,^43^ which could be due to *B. animalis* subsp. *lactis BL03x*, one of the components of the probiotic mixture that has been shown to produce valerate in *in vitro* fermentations.^38^ Our results revealed that the multistrain probiotic can be effective in reversing the increase of *Clostridia* UCG014 that occurred in the group of rats treated with PhIP + DSS, in agreement with the similar depletion observed by other authors for the genus *Clostridium* in the mucosal-adherent microbiota of AOM/IL10–/– mice after probiotic administration.^11^ In the present work, the probiotic was administered before the onset of inflammation, which has been associated with a prophylactic effect.^45^ This probiotic effect is time and dose dependent and may not be necessarily the same when it is administered after the onset of colonic inflammation.^19^

The microorganisms present in this commercial formula have been studied for their potential use as probiotics in the treatment and prevention of CRC.^19^ Based on the evidence showing that some lactobacilli strains mediate the conversion of PhIP into intermediates with reduced mutagenic potential^46^ and the role of *Bifidobacterium* in the maintenance of the intestinal barrier integrity;^47^ the chosen probiotic may be useful to revert shifts of the microbiota resulting from the administration of PhIP + DSS, despite the absence of modifications of its bacterial groups in feces.^39^ The authors suggest that this may be due to its effect on the regulation of the composition of beneficial and harmful bacteria^19^ and differential ability to colonize feces and colonic mucosa.^39^ In contrast to other studies, in our work, the mixed probiotic was administered with food instead of water. However, a reduction in markers related to inflammation has been observed in previous works independent of the administration method (orally gavage or dissolved in drinking water).^39,48^

There is strong evidence supporting the protective effect of dietary fiber consumption against CRC and their role as a modulator of the intestinal microbiota.^13^ In spite of this and to the best of our knowledge, no previous studies have approached the consumption of fiber in the context of an animal model treated with PhIP + DSS. In our study, the administration of an isocaloric diet enriched in fiber (6% fiber) to rats treated with PhIP + DSS reduced damage to the colonic mucosa by means of reducing crypt loss and diffuse inflammation, and by a nonsignificant partial counteracting effect on decreased colon length. Consistent with our results, increased colon length and restoration of colonic mucosal damage have been reported in animals with reduced DSS colitis scores and a similar dietary fiber content (5%).^49^ Dietary fiber promoted shifts in the gut microbiota by increasing the alpha diversity and the relative abundance of *Ruminococcus* (*Ruminococcaceae* family). In addition, a reduced relative abundance of *Clostridia* UCG014 and an increase of taxa belonging to the phylum Bacteroidota such as *Alistipes* (*Rikenellaceae* family) and *Bacteroidaceae* and genus *Bacteroides* were found in the present work as compared to rats treated with PhIP + DSS following a standard diet. Low relative proportions of *Rikenellaceae* and *Ruminococcus* have been found after DSS exposure in previous studies,^50^ these microorganisms displaying negative associations with pro-inflammatory cytokine levels in colitis.^51^ In contrast, higher relative abundances of Bacteroidota and *Rikenellaceae* were noted in colitis models fed with similar dietary fiber content as those used by us (5%),^23,49^ whereas increased abundance of *Bacteoridaceae* and *Ruminocccaceae* were found in mice fed with a high-fiber diet (35%) or pectin supplementation for 1 week (10%).^52^ Among these, the species *Ruminococcus bromii* seems to play a pivotal role in the degradation of a subtype of fiber.^53^ The increased relative abundance of these families in animals fed with high-fiber diets has been associated with the remodeling of the gut microbiota and the restoration of the intestinal barrier^43^ and thus may partially explain the mechanisms by which fiber supplementation reduced colonic mucosal damage in a PhIP animal model. In addition to shifts in gut microbiota composition, reduced absorption of HCAs in the presence of fiber has been previously described,^4^ which may contribute to the protective effect of dietary fiber intake at the colonic level. Moreover, although the use of an animal model limits the extrapolation of the results obtained to humans, our findings contribute to supporting the benefits of probiotic supplementation on colon elongation and the beneficial effect of dietary fiber on reducing colonic mucosal damage, inflammation, and thus the risk of CRC, probably through the modulation of gut microbiota composition, among other mechanisms.

In the present work, the consumption of PhIP + DSS in a dose mimicking a high regular PhIP dietary intake in humans provoked damage to the colonic mucosa through erosion, inflammation, and crypt loss, accompanied by shifts in the composition of the gut microbiota profile toward higher abundances of pro-inflammatory taxa such as *Clostridia* UCG014. At the doses administered, fiber was more effective than the mixed probiotic in alleviating damage induced by PhIP + DSS to the intestinal mucosa, while the probiotic favored colon elongation compared to animals in the PhIP + DSS group without probiotic supplementation.

## Supporting Information Available

Body weights and food and water intake, histologic colonic mucosal damage induced by PhIP + DSS and counteracting effect by probiotic or fiber supplementation in each colon segment, diversity indices and microbial relative abundances along the study in the control group, PhIP + DSS group, probiotic group, and fiber group, posthoc analysis of the relative abundances of gut microbiota taxa that changed significantly across the study within each experimental group, and effect of probiotic or fiber supplementation on the relative abundance of gut microbiota after the administration of PhIP and DSS (PDF)

## Abbreviations used

ACF: Aberrant crypt foci
AEI: Spanish Agency of Research
ANOVA: analysis of the variance
AOM: azoxymethane
ASV: amplicon sequence variants
BCFA: branched chain fatty acids
CRC: colorectal cancer
DSS: sodium dextran sulfate
HCAs: heterocyclic aromatic amines
IARC: International Agency for Research on Cancer
NFκB: nuclear factor kappa B
PBS: phosphate-buffered saline
PhIP: 2-amino-1-methyl-6-phenylimidazo [4,5-*b*] pyridine
QIIME2: Quantitative Insights Into Microbial Ecology
SCFA: short chain fatty acids
