# Supplementary material for: Potential of Fiber and Probiotics to Fight Against the Effects of PhIP + DSS-Induced Carcinogenic Process of the Large Intestine
Source: J Agric Food Chem. 2024 Oct 29;72(45):25161–72. doi: 10.1021/acs.jafc.4c07366 (PMC11565705; doi:10.1021/acs.jafc.4c07366)
Supplement: Supplementary file 1 — jf4c07366_si_001.pdf [file jf4c07366_si_001.pdf]

Supporting information

Potential of Fiber and Probiotics to Fight Against the Effects of PhIP + DSS-Induced Carcinogenic Process of the Large Intestine.

Aida Zapico [a,d], Nuria Salazar [b,d], Silvia Arboleya [b,d], Carmen González del Rey [c], Elena Diaz [a], Ana Alonso [a], Miguel Gueimonde [b,d], Clara G. de los Reyes-Gavilán [b,d], Celestino Gonzalez [a] and Sonia González [a,d *]

[a] Department of Functional Biology, University of Oviedo, Oviedo 33006, Spain

[b]Department of Microbiology and Biochemistry of Dairy Products, Instituto de Productos Lácteos de Asturias (IPLA-CSIC), Villaviciosa 33300, Spain

[c] Anatomical Pathology Service, Central University Hospital of Asturias (HUCA), Oviedo 33011, Spain

[d] Diet, Microbiota and Health Group, Instituto de Investigación Sanitaria del Principado de Asturias (ISPA), Oviedo 33011, Spain

* Email: soniagsolares@uniovi.es. Phone: +34-958-104-209

Table S1. Body weights and food and water intake

	Control (n=11)	PhIP + DSS (n=11)	Probiotic (n=11)	Fiber (n=11)
Body weight (g)				
Initial	186.91 ± 28.73	188.82 ± 20.52	184.36 ± 24.13	192.91 ± 20.88
Gained	69.00 ± 13.30	68.73 ± 11.67	70.00 ± 15.98	53.82 ± 8.95*
Intake (g/d)				
Food	15.61 ± 4.09	15.86 ± 4.17	16.77 ± 4.58	16.83 ± 4.42
Water	21.35 ± 6.11	20.22 ± 5.66	20.01 ± 6.33	23.71 ± 7.63

Data is expressed as mean ± sd. Statistical analysis for pairwise comparisons of control vs PhIP + DSS group, PhIP + DSS vs probiotic group, and PhIP + DSS vs fiber group was performed through *T*-test (* $p < 0.05$). DSS, sodium dextran sulfate, and PhIP, 2-amino-1-methyl-6-phenylimidazo [4,5-b] pyridine.

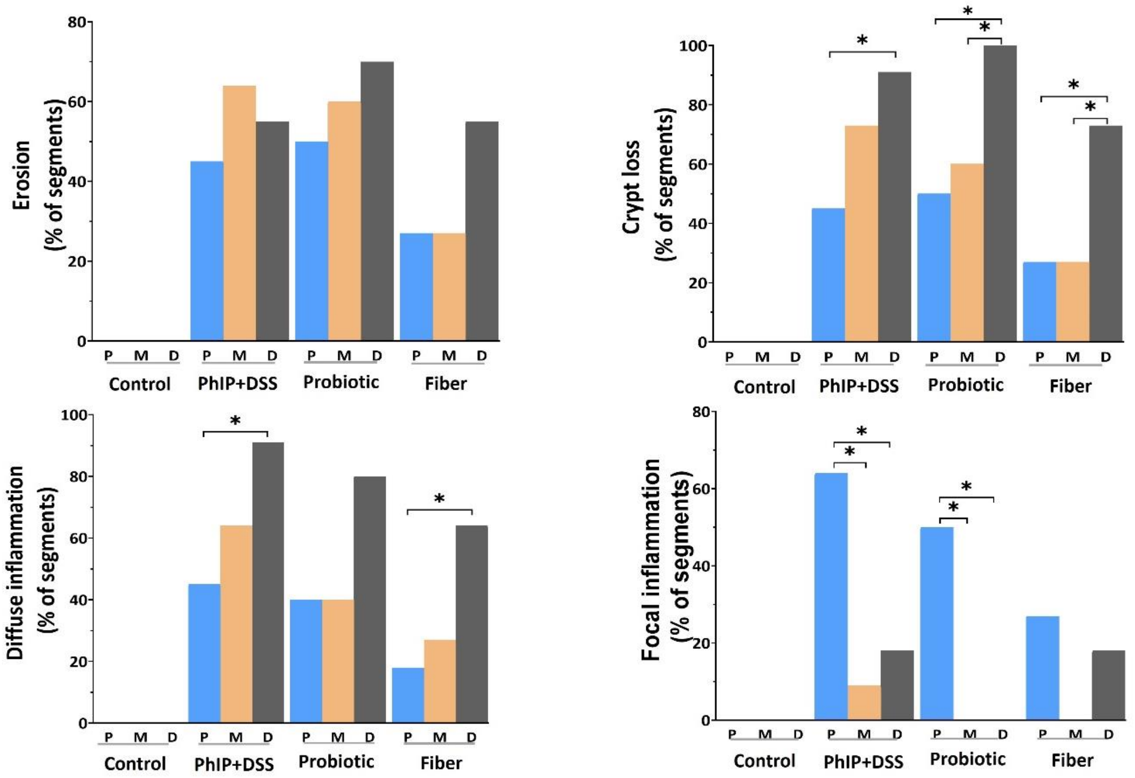

Figure S1. Histologic colonic mucosal damage induced by PhIP + DSS and counteracting effect by probiotic or fiber supplementation in each colon segment. Percentage of P, M and D colon segments presenting each histological feature (erosion, crypt loss, diffuse or focal inflammation) in each experimental group. Statistical analysis for pairwise comparisons (P vs M, P vs D, and M vs D) within each experimental group was performed with X^2 test (n= 11 for P, M and D in control, PhIP + DSS, probiotic and fiber groups) (* $p < 0.05$). D, distal; DSS, sodium dextran sulfate; M, mild; P, proximal; and, PhIP, 2-amino-1-methyl-6-phenylimidazo [4,5-b] pyridine.

Table S2. Diversity indices and microbial relative abundances along the study in the control group

	Baseline (n=8)	Pre treatment (n=8)	Post treatment (n=9)	End (n=9)	ANOVA (*p* value)
**Diversity**					
Shannon	6.94 ± 0.4	6.60 ± 0.71	6.41 ± 0.67	6.94 ± 0.46	0.162
**Taxa (%)**					
Bacillota	73.61 ± 0.07	0.79 ± 0.06	0.74 ± 0.08	0.77 ± 0.06	0.344
Lachnospiraceae	26.89 ± 0.16	0.24 ± 0.16	0.20 ± 0.15	0.23 ± 0.13	0.785
*Lachnospiraceae*_NK4A136_group	10.51 ± 0.09	0.12 ± 0.09	0.09 ± 0.08	0.09 ± 0.06	0.870
*Eubacterium_xylanophilum*_group	1.36 ± 0.02	0.02 ± 0.01	0.01 ± 0.01	0.01 ± 0.01	0.421
*Lachnospiraceae*_UCG006	1.11 ± 0.01	0.01 ± 0.02	0.01 ± 0.01	0.01 ± 0.01	0.629
Roseburia	1.01 ± 0.01	0.00 ± 0.00	0.00 ± 0.01	0.00 ± 0.00	0.467
Lactobacillaceae	9.96 ± 0.06	0.10 ± 0.05	0.09 ± 0.07	0.07 ± 0.05	0.624
Lactobacillus	9.96 ± 0.06	0.10 ± 0.05	0.09 ± 0.07	0.07 ± 0.05	0.624
Oscillospiraceae	7.00 ± 0.02	0.07 ± 0.03	0.07 ± 0.03	0.09 ± 0.03	0.284
Ruminococcaceae	6.45 ± 0.03	0.05 ± 0.02	0.05 ± 0.02	0.07 ± 0.02	0.259
Incertae_sedis	1.76 ± 0.01	0.01 ± 0.00	0.01 ± 0.01	0.01 ± 0.01	0.295
Peptostreptococcaceae	5.32 ± 0.04	0.07 ± 0.07	0.09 ± 0.08	0.07 ± 0.05	0.721
Romboutsia	5.22 ± 0.04	0.07 ± 0.07	0.09 ± 0.08	0.07 ± 0.05	0.721
*Clostridia*_UCG014_A	4.30 ± 0.02	0.08 ± 0.04	0.07 ± 0.05	0.06 ± 0.03	0.365
*Clostridia*_UCG014_B	4.30 ± 0.02	0.08 ± 0.04	0.07 ± 0.05	0.06 ± 0.03	0.365
Clostridiaceae	3.84 ± 0.03	0.09 ± 0.11	0.09 ± 0.10	0.06 ± 0.06	0.545
Clostridium_sensu_stricto_1	3.83 ± 0.03	0.09 ± 0.11	0.09 ± 0.10	0.06 ± 0.06	0.544
Erysipelotrichaceae	3.12 ± 0.03	0.04 ± 0.03	0.03 ± 0.02	0.06 ± 0.04	0.300
Turicibacter	2.38 ± 0.02	0.03 ± 0.03	0.03 ± 0.02	0.05 ± 0.05	0.262
Bacteroidota	22.90 ± 0.06	0.18 ± 0.06	0.22 ± 0.07	0.21 ± 0.06	0.440
Muribaculaceae	14.04 ± 0.04	0.12 ± 0.04	0.15 ± 0.05	0.14 ± 0.03	0.459
*Muribaculaceae*_A	13.89 ± 0.04	0.12 ± 0.04	0.15 ± 0.05	0.13 ± 0.03	0.468
Prevotellaceae	3.76 ± 0.04	0.02 ± 0.01	0.03 ± 0.02	0.03 ± 0.03	0.411
Alloprevotella	1.09 ± 0.02	0.01 ± 0.01	0.01 ± 0.01	0.01 ± 0.01	0.872
*Prevotellaceae*_NK3B31_group	2.10 ± 0.02	0.01 ± 0.00	0.01 ± 0.01	0.02 ± 0.02	0.218
Rikenellaceae	2.65 ± 0.01	0.02 ± 0.01	0.01 ± 0.01	0.02 ± 0.02	0.133
Alistipes	2.54 ± 0.01	0.02 ± 0.01	0.01 ± 0.01	0.01 ± 0.02	0.145
Bacteroidaceae	1.94 ± 0.01	0.01 ± 0.01	0.02 ± 0.02	0.01 ± 0.01	0.608
Bacteroides	1.94 ± 0.01	0.01 ± 0.01	0.02 ± 0.02	0.01 ± 0.01	0.608
Actinomycetota	1.95 ± 0.02	0.01 ± 0.01	0.01 ± 0.01	0.02 ± 0.01	0.802
Eggerthellaceae	1.70 ± 0.02	0.01 ± 0.01	0.01 ± 0.01	0.01 ± 0.01	0.740
Enterorhabdus	1.17 ± 0.01	0.01 ± 0.01	0.01 ± 0.01	0.01 ± 0.00	0.691

Data is expressed as mean relative abundance (%) ± SD. Only taxa with mean relative abundance greater than 1% are presented. No differences were found across the study according to one-way ANOVA ($p < 0.05$).

Table S3. Diversity indices and microbial relative abundances along the study in the PhIP + DSS group.

	Baseline (n=7)	Pre treatment (n=8)	Post treatment (n=5)	End (n=9)	ANOVA (*p* value)
**Diversity**					
Shannon	6.69 ± 0.72	6.70 ± 0.63	6.65 ± 0.73	6.70 ± 0.52	0.999
**Taxa (%)**					
Bacillota	70.62 ± 14.92	74.16 ± 11.13	73.93 ± 5.82	77.94 ± 11.53	0.669
Lachnospiraceae	29.51 ± 18.03	21.58 ± 11.74	18.16 ± 12.77	33.22 ± 14.82	0.218
*Eubacterium_xylanophilum*_group	1.24 ± 0.83	1.17 ± 0.85	0.64 ± 0.59	1.62 ± 0.64	0.158
*Lachnospiraceae*_NK4A136_group	13.61 ± 8.73	9.00 ± 4.49	7.61 ± 6.01	13.85 ± 9.89	0.357
*Lachnospiraceae*_UCG006	2.49 ± 2.86	1.51 ± 1.63	1.17 ± 0.52	3.68 ± 2.25	0.115
Oscillospiraceae	7.44 ± 1.22	6.27 ± 2.21	6.86 ± 2.34	9.33 ± 2.44	0.039*
Colidextribacter	0.73 ± 0.55	0.61 ± 0.34	0.64 ± 0.62	1.19 ± 0.69	0.160
Oscillibacter	1.09 ± 0.53	1.05 ± 0.90	0.50 ± 0.46	1.10 ± 0.56	0.367
UCG005	0.65 ± 0.31	0.70 ± 0.28	1.18 ± 0.64	0.68 ± 0.31	*0.090*
Lactobacillaceae	6.42 ± 5.24	9.07 ± 6.94	12.31 ± 11.28	7.94 ± 6.16	0.571
Lactobacillus	6.42 ± 5.24	9.07 ± 6.94	12.31 ± 11.28	7.94 ± 6.16	0.571
Ruminococcaceae	5.94 ± 3.07	7.41 ± 2.75	7.34 ± 3.56	6.78 ± 2.57	0.772
Incertae_sedis	0.91 ± 0.45	1.35 ± 0.42	3.29 ± 3.56	1.20 ± 0.42	*0.052*
Ruminococcus	2.6 ± 2.66	4.09 ± 2.44	2.65 ± 0.61	2.54 ± 0.85	0.327
*Clostridia*_UCG014_A	5.29 ± 3.56	6.42 ± 2.45	9.04 ± 3.37	3.93 ± 1.75	0.020*
*Clostridia*_UCG014_B	5.29 ± 3.56	6.42 ± 2.45	9.04 ± 3.37	3.93 ± 1.75	0.020*
Clostridiaceae	3.36 ± 1.50	7.81 ± 8.46	4.46 ± 5.52	2.22 ± 2.94	0.193
Clostridium_sensu_stricto_1	3.36 ± 1.50	7.81 ± 8.45	4.45 ± 5.53	2.21 ± 2.94	0.193
Peptostreptococcaceae	5.02 ± 2.67	6.03 ± 4.73	5.64 ± 5.45	6.10 ± 5.31	0.968
Romboutsia	5.00 ± 2.67	6.03 ± 4.73	5.63 ± 5.47	6.09 ± 5.31	0.967
Erysipelotrichaceae	2.87 ± 1.68	4.23 ± 4.92	3.01 ± 3.83	2.82 ± 1.79	0.807
Faecalibaculum	0.00 ± 0.00	0.08 ± 0.21	1.26 ± 2.78	0.01 ± 0.02	0.197
Turicibacter	2.70 ± 1.60	3.93 ± 4.9	1.21 ± 1.29	2.65 ± 1.88	0.466
Monoglobaceae	0.35 ± 0.40	0.62 ± 0.42	1.16 ± 0.83	0.39 ± 0.36	0.034*
Monoglobus	0.35 ± 0.40	0.62 ± 0.42	1.16 ± 0.83	0.39 ± 0.36	0.034*
*Eubacterium_coprostanoligenes*_group	0.90 ± 0.47	1.03 ± 0.68	1.16 ± 0.53	1.02 ± 0.76	0.926
*Eubacterium_coprostanoligenes*_group_A	0.90 ± 0.47	1.03 ± 0.68	1.16 ± 0.53	1.02 ± 0.76	0.926
Bacteroidota	26.76 ± 14.43	22.90 ± 11.63	21.60 ± 8.40	19.57 ± 11.02	0.682
Muribaculaceae	18.62 ± 14.61	14.92 ± 6.64	15.70 ± 4.59	14.28 ± 10.29	0.844
*Muribaculaceae*_A	18.46 ± 14.58	14.76 ± 6.60	15.56 ± 4.54	14.10 ± 10.22	0.841
Prevotellaceae	3.12 ± 1.99	1.81 ± 1.53	2.80 ± 4.41	1.46 ± 0.90	0.444
*Prevotellaceae*_NK3B31_group	1.58 ± 1.13	0.88 ± 0.54	1.21 ± 2.2	0.98 ± 0.81	0.674
Rikenellaceae	2.32 ± 1.30	2.55 ± 2.48	0.75 ± 0.49	0.70 ± 0.43	0.038*
Alistipes	2.26 ± 1.28	2.44 ± 2.36	0.64 ± 0.51	0.69 ± 0.42	0.035*
Bacteroidaceae	1.76 ± 0.80	2.79 ± 4.09	1.88 ± 1.15	2.60 ± 2.34	0.843
Bacteroides	1.76 ± 0.80	2.79 ± 4.09	1.88 ± 1.15	2.60 ± 2.34	0.843
Actinomycetota	1.31 ± 1.08	1.59 ± 0.93	2.61 ± 2.43	1.11 ± 0.93	0.241
Eggerthellaceae	1.09 ± 1.09	1.35 ± 0.92	2.12 ± 1.87	0.95 ± 0.82	0.322
Enterorhabdus	0.67 ± 0.65	0.90 ± 0.63	1.59 ± 1.41	0.64 ± 0.56	0.180
Pseudomonadota	0.58 ± 0.72	0.60 ± 0.96	1.28 ± 2.20	0.14 ± 0.16	0.330

Data is expressed as mean relative abundance (%) ± SD. Only taxa with mean relative abundance greater than 1% are presented. Statistical analysis for differences across the study was performed by one-way ANOVA (* $p < 0.05$). DSS, sodium dextran sulfate, and PhIP, 2-amino-1-methyl-6-phenylimidazo [4,5-b] pyridine

Table S4. Diversity indices and microbial relative abundances along the study in the probiotic group.

	Baseline (n=8)	Pre treatment (n=7)	Post treatment (n=4)	End (n=7)	ANOVA (*p* value)
**Diversity**					
Shannon	6.69 ± 0.52	6.37 ± 1.23	6.02 ± 0.43	7.11 ± 0.53	0.145
**Taxa (%)**					
Bacillota	71.33 ± 11.48	78.14 ± 11.25	64.87 ± 19.77	74.22 ± 6.25	0.354
Lachnospiraceae	26.80 ± 13.34	24.81 ± 18.54	21.25 ± 27.71	28.88 ± 10.52	0.902
*Eubacterium_xylanophilum*_group	1.92 ± 1.52	1.65 ± 1.45	0.19 ± 0.19	1.31 ± 0.39	0.132
*Lachnospiraceae*_NK4A136_group	12.48 ± 7.87	11.69 ± 9.98	7.90 ± 8.19	10.20 ± 3.77	0.786
*Lachnospiraceae*_UCG006	2.04 ± 1.55	0.82 ± 0.64	1.20 ± 0.95	2.98 ± 1.79	0.041*
Oscillospiraceae	8.57 ± 4.40	7.40 ± 3.83	8.81 ± 3.81	9.51 ± 2.79	0.773
Oscillibacter	0.93 ± 0.62	0.87 ± 0.96	0.59 ± 0.76	1.10 ± 0.39	0.714
UCG005	1.45 ± 2.21	0.70 ± 0.31	0.48 ± 0.22	0.71 ± 0.37	0.539
Lactobacillaceae	6.93 ± 4.82	10.67 ± 7.33	8.22 ± 6.37	10.84 ± 6.01	0.559
Lactobacillus	6.93 ± 4.82	10.67 ± 7.33	8.22 ± 6.37	10.84 ± 6.01	0.560
Ruminococcaceae	5.78 ± 1.99	4.66 ± 1.85	3.97 ± 1.25	6.20 ± 2.19	0.227
Incertae_Sedis	0.93 ± 0.47	0.87 ± 0.24	0.99 ± 0.60	1.02 ± 0.52	0.941
Ruminococcus	2.53 ± 1.20	2.34 ± 1.57	1.76 ± 0.21	2.39 ± 0.90	0.751
*Clostridia*_UCG014_A	5.29 ± 2.64	3.15 ± 1.55	2.50 ± 1.63	2.96 ± 0.52	0.043*
*Clostridia*_UCG014_*B*	5.29 ± 2.64	3.15 ± 1.55	2.50 ± 1.63	2.96 ± 0.52	0.043*
Clostridiaceae	5.35 ± 7.30	11.21 ± 17.18	3.81 ± 2.70	1.70 ± 1.14	0.352
Clostridium_sensu_stricto_1	5.34 ± 7.29	11.21 ± 17.18	3.81 ± 2.70	1.69 ± 1.15	0.352
Peptostreptococcaceae	5.24 ± 3.81	6.64 ± 5.71	7.76 ± 6.21	4.67 ± 3.73	0.708
Romboutsia	5.22 ± 3.82	6.62 ± 5.73	7.76 ± 6.21	4.67 ± 3.73	0.710
Erysipelotrichaceae	1.93 ± 1.26	1.94 ± 0.88	3.67 ± 4.20	2.20 ± 1.88	0.520
Turicibacter	1.63 ± 1.31	1.44 ± 0.99	3.46 ± 4.21	1.98 ± 1.74	0.426
Bacteroidota	24.75 ± 10.28	17.5 ± 9.67	32.02 ± 20.64	23.32 ± 5.85	0.252
Bacteroidaceae	1.86 ± 1.01	2.61 ± 2.45	3.78 ± 3.13	3.66 ± 2.17	0.337
Bacteroides	1.86 ± 1.01	2.61 ± 2.45	3.78 ± 3.13	3.66 ± 2.17	0.337
Muribaculaceae	17.87 ± 7.49	11.48 ± 6.00	24.40 ± 17.66	16.32 ± 3.82	0.147
Muribaculaceae_A	17.70 ± 7.43	11.37 ± 5.97	24.27 ± 17.62	16.09 ± 3.80	0.146
Prevotellaceae	3.23 ± 2.72	1.05 ± 0.65	3.11 ± 2.70	1.96 ± 2.00	0.227
*Prevotellaceae*_NK3B31_group	2.12 ± 1.86	0.66 ± 0.47	2.51 ± 2.57	0.82 ± 0.75	0.104
Rikenellaceae	1.24 ± 0.54	1.91 ± 2.50	0.47 ± 0.15	0.85 ± 0.38	0.337
Alistipes	1.20 ± 0.54	1.86 ± 2.45	0.46 ± 0.15	0.84 ± 0.37	0.350
Actinomycetota	1.84 ± 1.52	1.31 ± 0.85	1.96 ± 0.89	1.63 ± 0.60	0.729
Eggerthellaceae	1.69 ± 1.48	1.05 ± 0.92	1.60 ± 1.01	1.45 ± 0.57	0.700
Enterorhabdus	1.09 ± 0.98	0.62 ± 0.54	0.78 ± 0.49	0.70 ± 0.35	0.554
Verrucomicrobiota	1.21 ± 2.14	0.44 ± 0.70	0.09 ± 0.04	0.16 ± 0.12	0.350
Akkermansiaceae	1.21 ± 2.14	0.44 ± 0.70	0.09 ± 0.04	0.16 ± 0.12	0.350
Akkermansia	1.21 ± 2.14	0.44 ± 0.70	0.09 ± 0.04	0.16 ± 0.12	0.350
Pseudomonadota	0.36 ± 0.44	2.14 ± 3.14	0.62 ± 1.00	0.32 ± 0.37	0.176
Enterobacteriaceae	0.12 ± 0.18	1.76 ± 3.12	0.24 ± 0.41	0.09 ± 0.19	0.199
EscherichiaShigella	0.02 ± 0.03	1.62 ± 3.19	0.24 ± 0.41	0.08 ± 0.16	0.256

Data is expressed as mean relative abundance (%) ± SD. Only taxa with mean relative abundance greater than 1% are presented. Statistical analysis for differences across the study was performed by one-way ANOVA (* $p < 0.05$).

Table S5. Diversity indices and microbial relative abundances along the study in the fiber group.

	Baseline (n=7)	Pre treatment (n=8)	Post treatment (n=8)	End (n=9)	ANOVA (*p* value)
**Diversity**					
Shannon	6.56 ± 0.69	6.09 ± 0.71	7.15 ± 0.36	6.96 ± 0.56	0.006*
**Taxa (%)**					
Bacillota	77.62 ± 6.54	76.13 ± 9.46	66.81 ± 9.25	77.05 ± 10.65	*0.087*
Lachnospiraceae	21.32 ± 14.43	23.96 ± 17.84	24.31 ± 12.44	32.12 ± 17.29	0.542
*Eubacterium_xylanophilum*_group	1.34 ± 0.60	1.10 ± 0.88	0.67 ± 0.36	0.85 ± 0.49	0.182
Lactobacillaceae	12.11 ± 6.16	9.02 ± 7.55	7.55 ± 4.80	9.11 ± 5.13	0.529
Lactobacillus	12.11 ± 6.16	9.02 ± 7.55	7.55 ± 4.80	9.11 ± 5.13	0.529
Clostridiaceae	8.62 ± 9.01	12.31 ± 13.30	2.66 ± 1.40	3.90 ± 4.37	*0.093*
Clostridium_sensu_stricto_1	8.61 ± 9.01	12.30 ± 13.30	2.57 ± 1.51	3.90 ± 4.37	*0.091*
*Lachnospiraceae*_NK4A136_group	8.00 ± 6.10	14.15 ± 11.31	8.69 ± 5.48	12.41 ± 7.41	0.377
*Lachnospiraceae*_UCG006	1.17 ± 1.00	0.87 ± 0.65	1.71 ± 1.17	2.64 ± 1.44	0.016*
Peptostreptococcaceae	7.73 ± 5.23	9.63 ± 7.05	5.17 ± 2.70	6.62 ± 7.55	0.515
Romboutsia	7.58 ± 5.14	9.56 ± 7.12	5.13 ± 2.73	6.59 ± 7.57	0.526
Oscillospiraceae	6.89 ± 2.93	6.07 ± 2.94	8.79 ± 2.91	8.35 ± 2.81	0.227
Colidextribacter	0.51 ± 0.38	0.84 ± 0.69	0.94 ± 0.56	1.26 ± 0.84	0.178
Oscillibacter	0.62 ± 0.47	1.00 ± 0.95	0.65 ± 0.26	0.79 ± 0.35	0.546
Ruminococcaceae	6.20 ± 0.74	4.50 ± 3.05	6.26 ± 1.51	6.32 ± 1.37	0.176
Incertae_Sedis	1.29 ± 0.67	1.60 ± 1.54	1.48 ± 0.69	1.33 ± 0.53	0.908
Ruminococcus	2.61 ± 1.38	1.32 ± 0.64	2.73 ± 1.51	3.21 ± 1.56	0.045*
*Clostridia*_UCG014_A	5.55 ± 2.34	3.09 ± 2.24	4.00 ± 2.53	3.05 ± 2.28	0.159
*Clostridia*_UCG014_B	5.55 ± 2.34	3.09 ± 2.24	4.00 ± 2.53	3.05 ± 2.28	0.159
Erysipelotrichaceae	1.93 ± 1.31	3.31 ± 1.46	2.18 ± 1.27	2.65 ± 1.87	0.319
Turicibacter	1.70 ± 1.37	2.63 ± 1.99	2.02 ± 1.30	2.59 ± 1.85	0.648
*Eubacterium_coprostanoligenes*_group	1.10 ± 0.78	0.60 ± 0.35	0.88 ± 0.46	0.85 ± 0.39	0.316
*Eubacterium_coprostanoligenes*_group_A	1.10 ± 0.78	0.60 ± 0.35	0.88 ± 0.46	0.85 ± 0.39	0.316
Bacteroidota	18.44 ± 7.07	19.28 ± 7.03	29.68 ± 9.50	21.53 ± 10.40	*0.065*
Muribaculaceae	14.05 ± 4.17	14.09 ± 3.63	18.29 ± 7.39	11.9 ± 4.91	0.115
*Muribaculaceae*_A	13.87 ± 4.07	14.01 ± 3.62	18.12 ± 7.33	11.78 ± 4.88	0.115
Bacteroidaceae	1.67 ± 1.80	2.60 ± 3.35	6.09 ± 3.88	4.65 ± 3.01	0.043*
Bacteroides	1.67 ± 1.80	2.60 ± 3.35	6.09 ± 3.88	4.65 ± 3.01	0.043*
Rikenellaceae	1.34 ± 0.94	0.97 ± 0.97	1.63 ± 0.98	0.80 ± 0.24	0.189
Alistipes	1.31 ± 0.95	0.94 ± 0.95	1.58 ± 0.95	0.77 ± 0.23	0.200
Prevotellaceae	1.01 ± 0.96	0.91 ± 0.64	2.42 ± 2.48	3.15 ± 4.09	0.242
*Prevotellaceae*_UCG001	0.15 ± 0.17	0.14 ± 0.09	0.92 ± 1.87	2.04 ± 3.33	0.197
Tannerellaceae	0.33 ± 0.22	0.65 ± 0.51	1.12 ± 0.70	0.98 ± 0.71	*0.061*
Parabacteroides	0.33 ± 0.22	0.65 ± 0.51	1.1 ± 0.67	0.97 ± 0.71	*0.060*
Actinomycetota	2.13 ± 1.40	1.30 ± 0.61	1.19 ± 0.52	0.74 ± 0.41	0.016*
Eggerthellaceae	1.89 ± 1.34	0.90 ± 0.68	0.87 ± 0.52	0.53 ± 0.28	0.012*
Enterorhabdus	1.24 ± 0.89	0.59 ± 0.42	0.58 ± 0.37	0.35 ± 0.20	0.013*
Pseudomonadota	1.21 ± 1.45	2.60 ± 3.50	1.72 ± 1.43	0.38 ± 0.52	0.175
Enterobacteriaceae	0.10 ± 0.17	1.87 ± 3.16	0.34 ± 0.35	0.11 ± 0.19	0.101
EscherichiaShigella	0.02 ± 0.05	1.75 ± 3.23	0.12 ± 0.20	0.03 ± 0.05	0.112

Data is expressed as mean relative abundance (%) ± SD. Only taxa with mean relative abundance greater than 1% are presented. Statistical analysis for differences across the study was performed by one-way ANOVA (* $p < 0.05$).

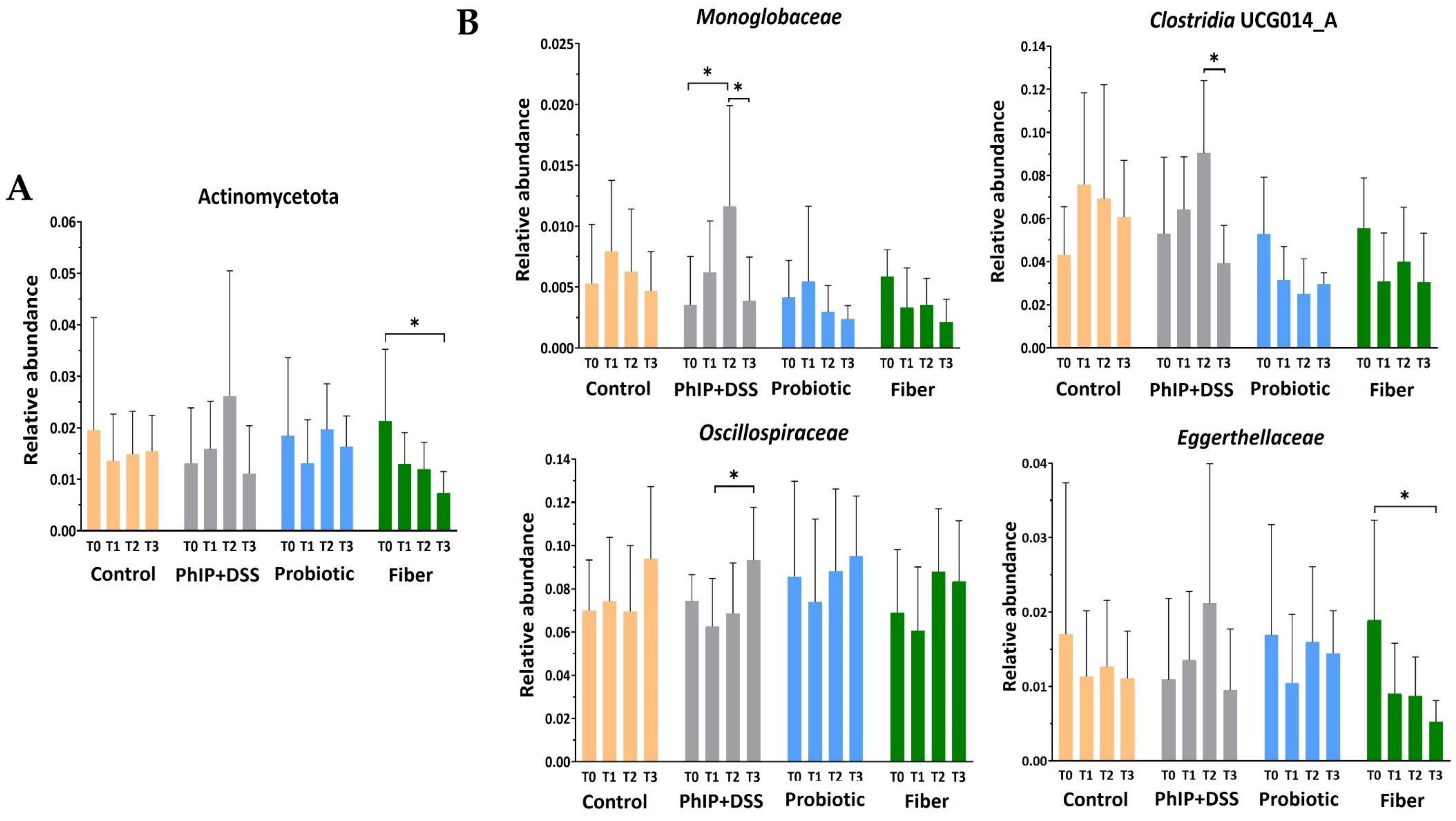
A
Actinomycetota
Relative abundance
0.06
0.05
0.04
0.03
0.02
0.01
0.00
*
T0 T1 T2 T3
Control
PhIP+DSS
Probiotic
Fiber
B
Monoglobaceae
0.025
0.020
0.015
0.010
0.005
0.000
Clostridia UCG014_A
0.14
0.12
0.10
0.08
0.06
0.04
0.02
0.00
Oscillospiraceae
Eggerthellaceae
0.04
0.03
0.02
0.01
0.00

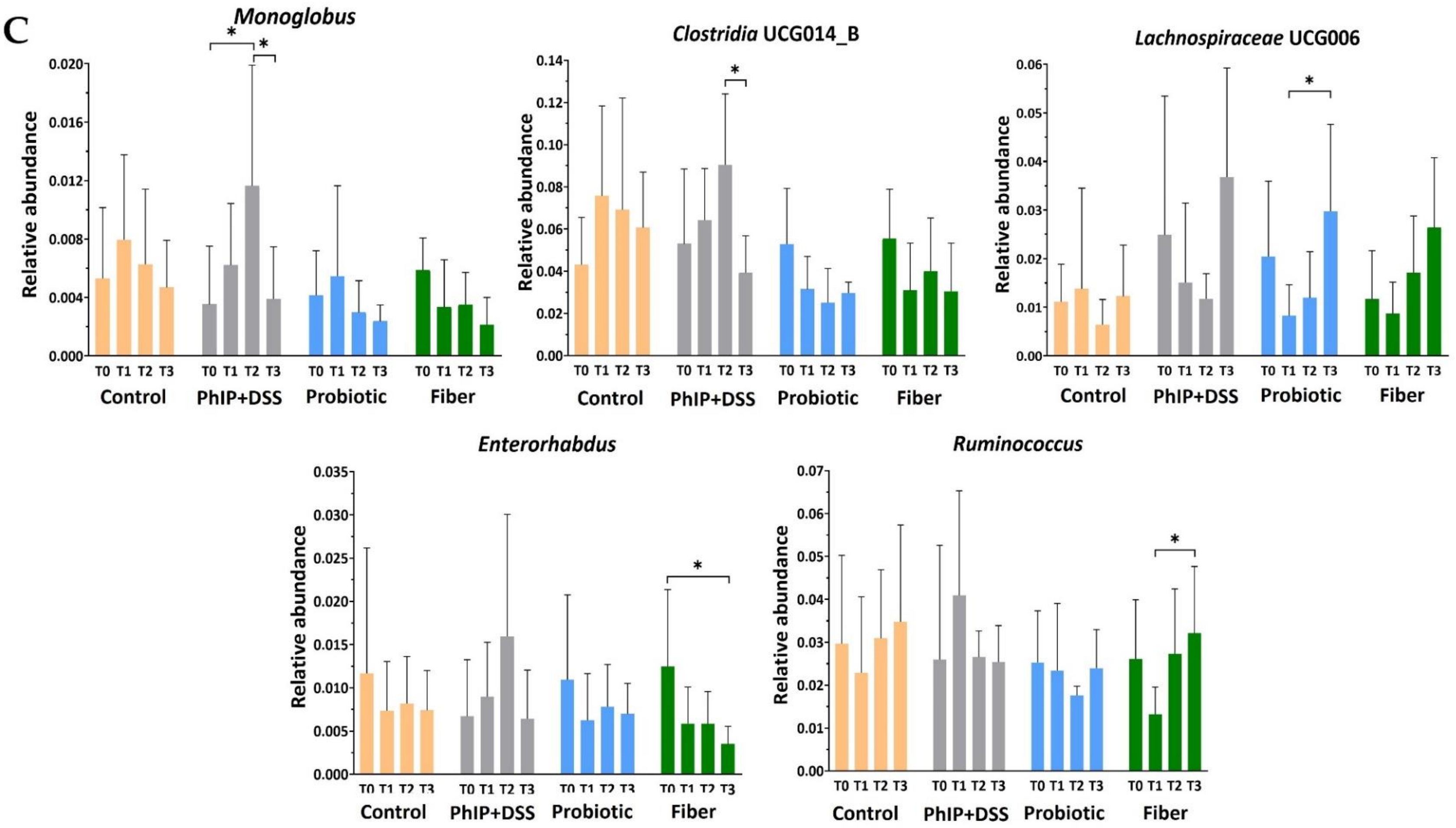

Figure S2. Post-hoc analysis of the relative abundances of gut microbiota taxa that changed significantly across the study within each experimental group. Bar plots represent mean relative abundance ± SD at the level of (A) phylum, (B) family and (C) genus. (A-C) Tukey's post-hoc HSD statistical analysis for pairwise comparisons across the study for each experimental group was performed (Control n=8 n= 8 n =9 and n=9; PhIP + DSS n=7 n= 8 n =5 and n=9; Probiotic n=8 n= 7 n =4 and n=7; and Fiber n =7 n= 8 n =8 and n=9; at T0, T1, T2 and T3 respectively) (* $p < 0.05$). DSS, sodium dextran sulfate; PhIP, 2-amino-1-methyl-6-phenylimidazo [4,5-b] pyridine; T0, baseline; T1, pre-treatment; T2, post-treatment; T3, end of the study

A

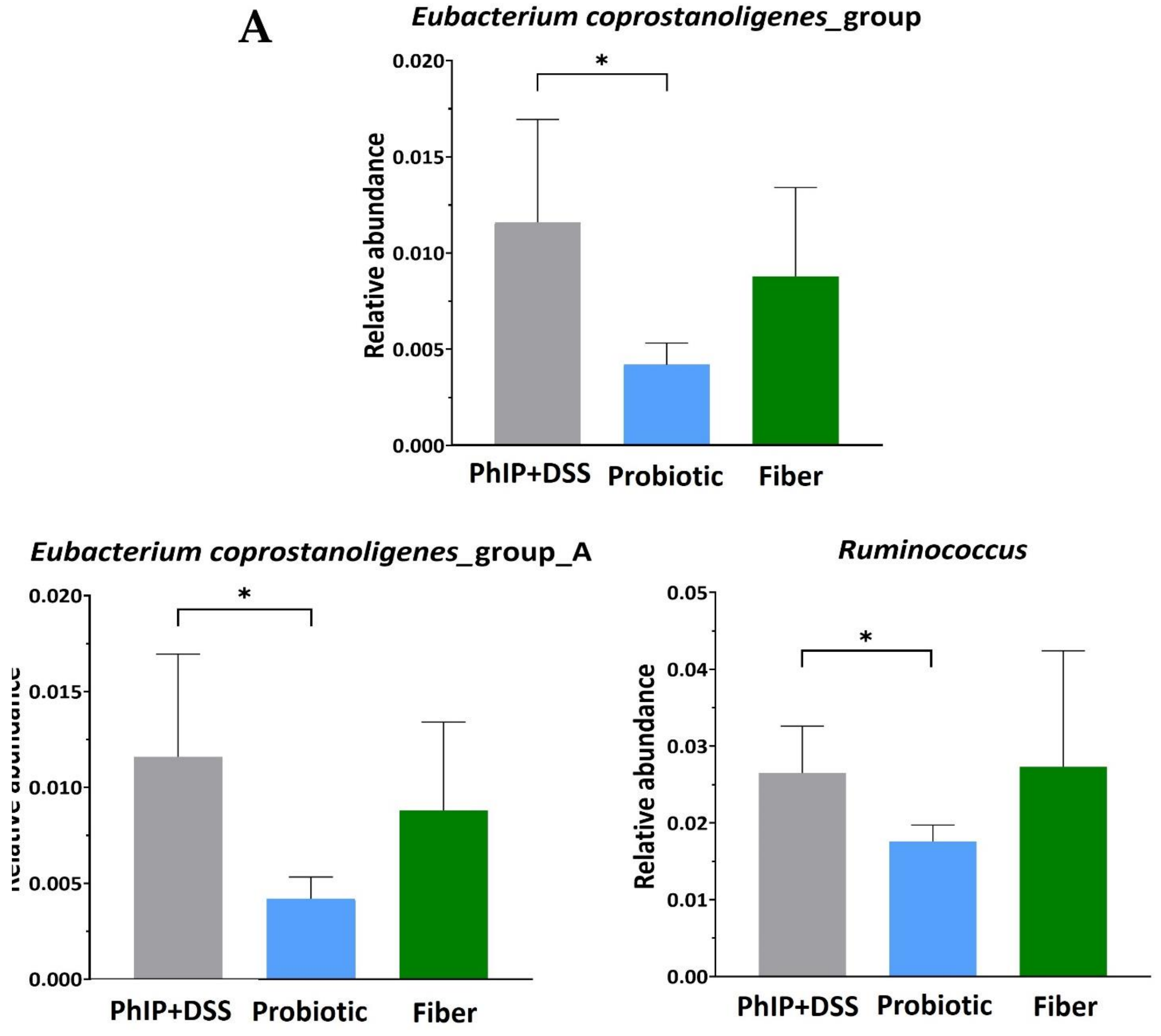
Eubacterium coprostanoligenes_group
Relative abundance
0.020
0.015
0.010
0.005
0.000
*
PhIP+DSS
Probiotic
Fiber
Eubacterium coprostanoligenes_group_A
0.020
0.015
0.010
0.005
0.000
*
PhIP+DSS
Probiotic
Fiber
Ruminococcus
Relative abundance
0.05
0.04
0.03
0.02
0.01
0.00
*
PhIP+DSS
Probiotic
Fiber

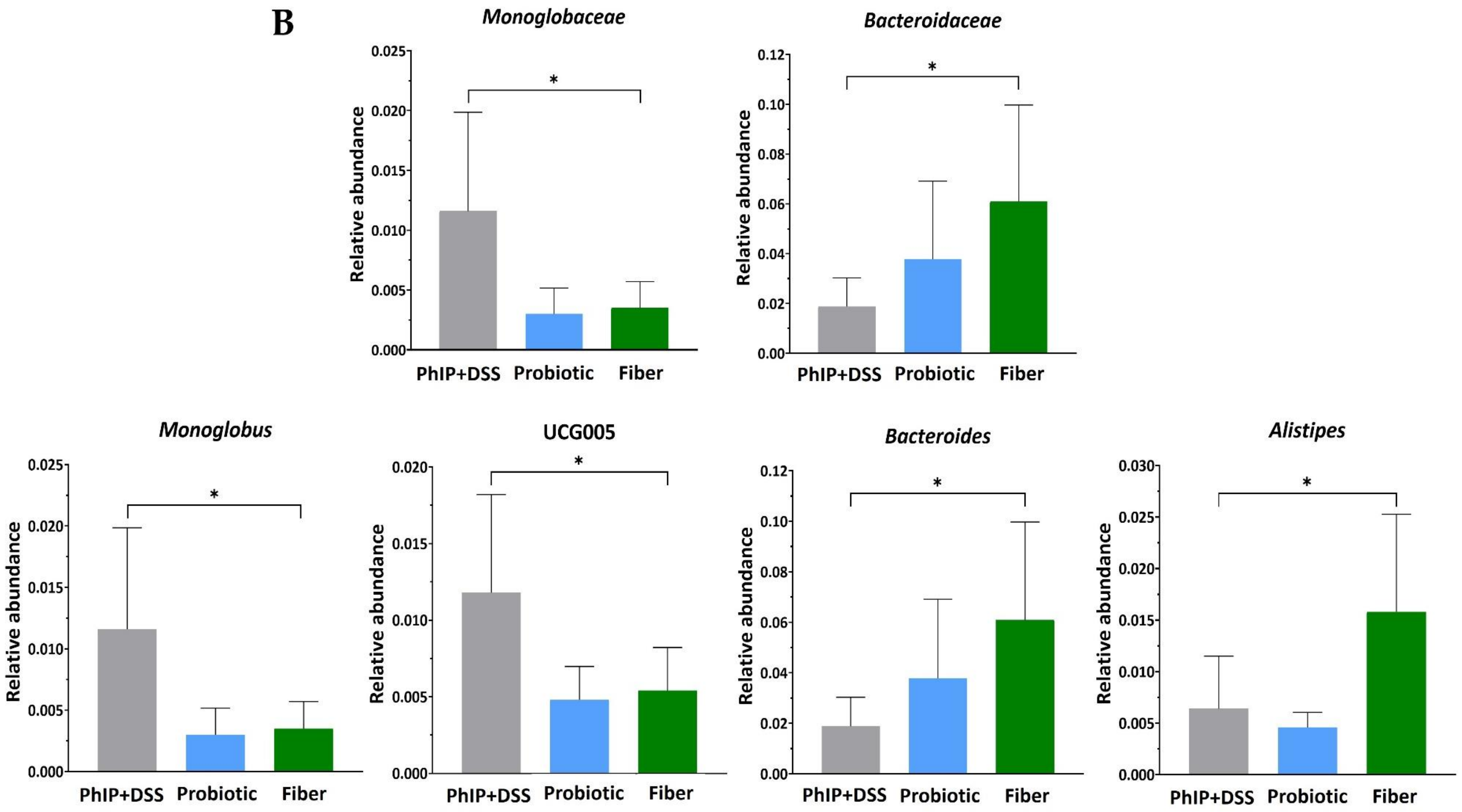

Figure S3. Effect of probiotic or fiber supplementation on the relative abundance of gut microbiota after the administration of PhIP and DSS. Effect of dietary supplementation with (A) probiotic or (B) fiber. Bar plots represent mean relative abundance ± SD. Statistical analysis for pairwise comparisons of (A) PhIP + DSS vs probiotic group, and (B) PhIP + DSS vs fiber group at post-treatment was performed by *T*-test (PhIP + DSS n= 5, Probiotic n= 4, Fiber n=8) (* $p < 0.05$). Only taxa with a mean relative abundance greater than 1% showing significant differences are shown. DSS, sodium dextran sulfate, and PhIP, 2-amino-1-methyl-6-phenylimidazo [4,5-b] pyridine.
